# Mechanism of the Ca^2+^-Dependent Interaction between S100A4 and Tail Fragments of Nonmuscle Myosin Heavy Chain IIA

**DOI:** 10.1016/j.jmb.2010.11.036

**Published:** 2011-01-28

**Authors:** Sandip K. Badyal, Jaswir Basran, Nina Bhanji, Ju Hwan Kim, Alap P. Chavda, Hyun Suk Jung, Roger Craig, Paul R. Elliott, Andrew F. Irvine, Igor L. Barsukov, Marina Kriajevska, Clive R. Bagshaw

**Affiliations:** 1 Department of Biochemistry, University of Leicester, Henry Wellcome Building, Lancaster Road, Leicester LE1 9HN, UK; 2 Department of Cell Biology, University of Massachusetts Medical School, Worcester, MA 01655, USA; 3 School of Biological Sciences, University of Liverpool, BioSciences Building, Crown Street, Liverpool L69 7ZB, UK; 4 Department of Cancer Studies and Molecular Medicine, University of Leicester, Robert Kilpatrick Clinical Sciences Building, Leicester LE2 7LX, UK

**Keywords:** NM-MHC IIA, nonmuscle myosin heavy chain IIA, ITC, isothermal titration calorimetry, EGTA, ethylene glycol bis(*b*-aminoethyl ether) *N*,*N*′-tetraacetic acid, HSQC, heteronuclear single-quantum coherence, FRAP, fluorescence recovery after photobleaching, GFP, green fluorescent protein, TIRF, total internal reflection fluorescence, transient kinetics, EF hand, fluorescence, isothermal titration calorimetry, stopped flow

## Abstract

The interaction between the calcium-binding protein S100A4 and the C-terminal fragments of nonmuscle myosin heavy chain IIA has been studied by equilibrium and kinetic methods. Using site-directed mutants, we conclude that Ca^2+^ binds to the EF2 domain of S100A4 with micromolar affinity and that the *K*_d_ value for Ca^2+^ is reduced by several orders of magnitude in the presence of myosin target fragments. The reduction in *K*_d_ results from a reduced dissociation rate constant (from 16 s^− 1^ to 0.3 s^− 1^ in the presence of coiled-coil fragments) and an increased association rate constant. Using peptide competition assays and NMR spectroscopy, we conclude that the minimal binding site on myosin heavy chain IIA corresponds to A1907-G1938; therefore, the site extends beyond the end of the coiled-coil region of myosin. Electron microscopy and turbidity assays were used to assess myosin fragment filament disassembly by S100A4. The latter assay demonstrated that S100A4 binds to the filaments and actively promotes disassembly rather than just binding to the myosin monomer and displacing the equilibrium. Quantitative modelling of these *in vitro* data suggests that S100A4 concentrations in the micromolar region could disassemble myosin filaments even at resting levels of cytoplasmic [Ca^2+^]. However, for Ca^2+^ transients to be effective in further promoting dissociation, the elevated Ca^2+^ signal must persist for tens of seconds. Fluorescence recovery after photobleaching of A431/SIP1 cells expressing green fluorescent protein–myosin IIA, immobilised on fibronectin micropatterns to control stress fibre location, yielded a recovery time constant of around 20 s, consistent with *in vitro* data.

## Graphical Abstract

**Figure:**
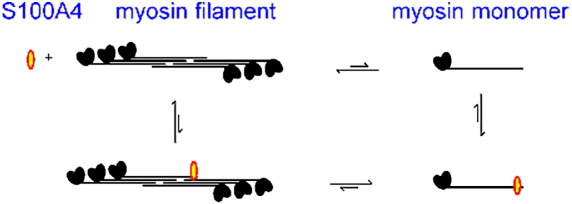

**Research Highlights:**

► Ca^2+^ binds preferentially to the EF2 domain of S100A4. ► The minimal S100A4 binding site on myosin IIA includes residues A1907-G1938. ► Aggregates of a myosin tail fragment dissociate via an S100A4–aggregate complex. ► In the presence of Ca^2+^, S100A4 binds the monomeric myosin tail with nanomolar affinity. ► Myosin could interact with S100A4 at resting cytoplasmic [Ca^2+^].

## Introduction

Over the last two decades, there has been much interest in the role of the S100 Ca^2^^+^-binding protein family in the regulation of cellular events.^1,2^ Identifying the specific targets of each of the 24 members^3^ and deducing their mechanisms of action *in vitro* and *in vivo* remain to be a challenge. This is exemplified by S100A4 (also known as Mts1, metastasin, p9Ka, pEL98, calvasculin, and Fsp-1), which has been implicated in a wide range of cellular events and numerous diseases and binds to several different targets *in vitro*.^4–6^ In particular, following early work,^7–9^ there have been many studies on the interaction of S100A4 and nonmuscle myosin heavy chain IIA (NM-MHC IIA). In the presence of Ca^2+^, S100A4 promotes the disassembly of NM-MHC IIA filaments and inhibits the assembly of monomeric NM-MHC IIA into filaments.^10^ Using NM-MHC IIA rods as a model system, Li *et al.* concluded that S100A4 bound tightly to the monomeric myosin tail to increase its critical concentration, thus inhibiting filament formation.^11^ At 20 mM NaCl, S100A4 was ineffective at dissociating rod filaments, but it did bind to rods with micromolar affinity.^11^ However, it remains an open question as to whether S100A4 depolymerises myosin or rod filaments just by sequestering the monomeric species and allowing the monomer–polymer equilibrium to readjust, or whether S100A4 binds to the myosin rod region in filaments and actively promotes depolymerisation.

S100 proteins contain two EF-hand Ca^2+^ binding sites, but the N-terminal domain (EF1) is a pseudo EF-hand. It contains 14 residues—instead of the usual 12 residues—in the Ca^2+^ binding loop, and, apart from glutamate 33, backbone carbonyl residues provide Ca^2+^ binding ligands instead of side chains. Literature *K*_d_ values for Ca^2+^ binding to S100A4 show a disparate range from the micromolar region to the millimolar region.^12–18^ Garrett *et al.*^16^ and Malashkevich *et al.*^17^ determined two classes of site by various methods and considered the higher-affinity site to represent Ca^2+^ binding to the canonical EF2 hand, with the weaker site representing the pseudo EF1 hand. However, detailed studies on the related protein calbindin D9k indicated that both EF1 and EF2 sites bound Ca^2+^ with comparable affinities and that there was cooperativity between them.^19,20^ These results indicated that a pseudo EF1 site can potentially bind Ca^2+^ with a very high affinity (10^− 8^ M) and, therefore, the weaker site cannot be assumed to be EF1 without additional evidence. Interestingly, NMR titration studies on S100A4 indicated that the pseudo EF1 hand may bind Ca^2+^ more tightly than EF2, as some residues in the N-terminus were perturbed at lower Ca^2+^ ratios compared with those in the C-terminus.^15^

Apart from the different methods used to study Ca^2+^ binding, which have sensitivities over different concentration ranges, part of the variability in *K*_d_ values in the literature may arise from the self-association of S100A4. There is a general consensus^12,13,17,21,22^ that S100A4 exists predominantly as a dimer at micromolar concentrations (indeed, this can lead to difficulties in interpreting literature values for stoichiometries quoted as ‘moles of Ca^2+^ bound per mole of S100A4’ if the latter is not stated explicitly as being per monomer or per dimer). In the absence of Ca^2+^, monomeric S100A4 can be detected at protein concentrations below 2 μM.^21^ At concentrations exceeding 20 μM S100A4 in the presence of Ca^2+^, tetrameric and higher aggregates become significant.^17,21,22^ Secreted S100A4 exists in these higher-order states, possibly aided by cysteine disulphide cross-linking, to effect additional extracellular functions.^23^ S100A4 crystals, grown in the presence of Ca^2+^, revealed a tetrameric structure in which the C-terminal extension of one S100A4 monomer bound to the target binding site of an S100A4 monomer in the apposing dimer.^18^ While Ca^2+^ is not required for dimerisation or further oligomerisation, Ca^2+^ tends to stabilise higher-order quaternary structures; therefore, the measured Ca^2+^ affinity will depend on the protein concentration used. Thermodynamic coupling requires the higher-order oligomerisation states to bind Ca^2+^ more tightly. Here, we reexamine the Ca^2+^ binding properties of S100A4, including mutant E33Q and D63N constructs, which are compromised in the EF1 and EF2 Ca^2+^ binding loops, respectively.^24^

Comparison of the high-resolution NMR structure of S100A4 in the absence of Ca^2+^ (Protein Data Bank code 1M31^25^) with crystal structures obtained in the presence of Ca^2+^ (Protein Data Bank codes 3C1V,^18^ 2Q91,^17^ and 3CGA^26^) shows that, in common with other S100 proteins, Ca^2+^ binding to EF2 induces a 60° reorientation of helix III to expose a hydrophobic surface that constitutes at least part of the target binding site. In the absence of Ca^2+^, S100A4 shows a very low affinity for many of its targets,^2^ including the binding of NM-MHC IIA and its tail fragments.^7,8,11^ Thermodynamic linkage requires the target-bound form of S100A4 to bind Ca^2+^ more tightly than in the absence of a target protein.^14,16^ We extend these studies by examining the Ca^2^^+^-dependent kinetics of target binding and the kinetics of Ca^2+^ dissociation from S100A4 in the absence and in the presence of targets. These studies are pertinent to assessing the extent of interaction of S100A4 with NM-MHC IIA at resting cytoplasmic [Ca^2+^] and the changes induced by spikes, waves, or a more prolonged elevation of [Ca^2+^] on excitation. Previous reviews have commented on the apparent anomaly (the S100 dilemma^27^) that many S100 proteins bind Ca^2+^ in the 10–50 μM range, which is 2 orders of magnitude higher than the time-averaged cytoplasmic [Ca^2+^] of eukaryotic cells, and barely reach peak excitation.^2^

**Fig. 1:**
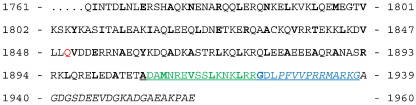
Sequence of C-terminal human NM-MHC IIA fragments and peptides. The His-tagged expressed fragments M200 and M111 contained an N-terminal fusion comprising residues MRGSHHHHHGS. The sequence shown corresponds to M200 (residues Q1761-E1960; native C-terminus). M111 starts at residue Q1850 (red). The M32 peptide (A1907-G1938) is underlined, M16^N^ (D1908-R1923) is shown in green, and M15^C^ (G1924-G1938) is shown in blue. Residues in the *a* and *d* heptad repeat positions of the coiled coil (generally hydrophobic) are shown in boldface. The nonhelical C-terminus region is shown in italics. Note that the numbering system is one residue shorter than that originally used by Kriajevska *et al.* and corresponds to the current nonmuscle myosin IIA database nomenclature (NCBI reference sequence NM_002473.4).^9^ Thus, M200 is equivalent to the Hmyo4-3B fragment.^9^ Note that the M200 and M111 preparations contain a V1913I substitution.

Our kinetic studies are also used to redefine the minimal binding fragment of the NM-MHC IIA tail (Fig. 1). Using a series of overlapping fragments and a blot overlay assay, Kriajevska *et al.* previously mapped the S100A4 binding site of nonmuscle myosin IIA to a 29-amino-acid region near the C-terminus (residues 1908–1936 in the current numbering system).^9^ Subsequently, Malashkevich *et al.* reported only around a 2-fold difference in binding constant between a shorter peptide comprising residues 1908–1923 (identical with M16^N^ in the present study) and longer fragments (residues 1851–1960 and 1893–1923), and they concluded that the former represented the minimal S100A4 binding site.^17^ Here we reexamine the properties of myosin-based peptides' binding to S100A4 and find that the fluorescein tag, as used by Malashkevich *et al.*, makes a large contribution to binding energy and that the binding site extends beyond the “minimal” region of residues 1908–1923.^17^

## Results

### Ca^2+^ binding to S100A4 and mutant constructs

**Fig. 2:**
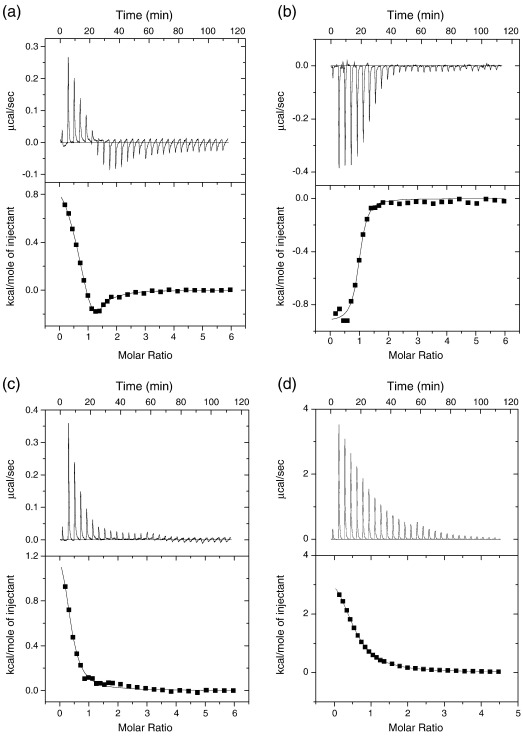
ITC records for Ca^2+^ binding to S100A4 constructs: (a) 75 μM wild type at 30 °C; (b) 75 μM wild type at 10 °C; (c) 75 μM E33Q construct at 30 °C; (d) 300 μM D63N construct at 30 °C. For mutant constructs data at 10 °C, see Fig. S1. The fitted lines in the bottom panels correspond to the data shown in Table 1. All preparations were dialysed against the same buffer of 20 mM NaCl, 10 mM Hepes, and 2 mM DTT (pH 7.5).

Malashkevich *et al.* investigated Ca^2+^ binding to S100A4 using isothermal titration calorimetry (ITC) and reported that, at 37 °C and 20 mM KCl, two classes of site with *K*_d_ values of 3.3 μM and 54 μM at positive and negative enthalpies, respectively, were apparent.^17^ We obtained comparable results at 30 °C (Fig. 2, Table 1) but found that the parameters of the second site were not well defined. Under our standard buffer conditions at 20 °C, the enthalpy changes were smaller, and the second site was not evident. At 10 °C, the first site became associated with negative enthalpy but retained a similar *K*_d_ value, while the second site remained undetectable. While this might be caused by a change in affinity, it is possible that the enthalpy change for the second site is small and masked by the first. In some titrations at 10 °C, the ITC profile indicated positive cooperativity. The variability in these titrations may arise from differences in the small amounts of contaminant Ca^2+^ present at the start of the titration. Titration against ethylene glycol bis(*b*-aminoethyl ether) *N*,*N*′-tetraacetic acid (EGTA) indicated that about 20% of the S100A4 had prebound Ca^2+^ present. The measured stoichiometry of the higher-affinity site was 0.8–0.9 Ca^2+^ per monomer (Table 1), which, after allowance for the prebound Ca^2+^, indicates that the high-affinity site corresponds to a single EF hand. This conclusion, however, depends on the accuracy of the S100A4 concentration determination (see Discussion).

In an attempt to assign the measured affinities to the EF1 and EF2 sites, we examined S100A4 constructs in which an acidic side chain that contributes to each of the Ca^2+^ binding sites was mutated to its corresponding amide (E33Q and D63N), as performed previously.^24^ The E33Q construct showed some similarities with the wild-type S100A4 in that it bound Ca^2+^ in the region of 1–10 μM, with an indication of cooperativity at 10 °C; however, the stoichiometry was reduced at 30 °C, suggesting that the protein may be less stable (Fig. 2c; Fig. S1a). The D63N mutant bound Ca^2+^ much more weakly (Fig. 2d; Fig. S1b) and required the protein concentration to be increased 4-fold for ITC measurements. A *K*_d_ of 65–70 μM and positive enthalpy were recorded at both 10 °C and 30 °C, but the change in enthalpy with temperature was similar to that of wild type. The interpretation of these mutant constructs is not straightforward as it appears that there is communication between the EF1 domain and the EF2 domain. However, the simplest explanation is that the higher-affinity site observed by ITC with the wild type corresponds to EF2 because this site is retained in the E33Q mutant. In the D63N construct, the observed weaker Ca^2+^ binding could represent binding to EF1, but the size and temperature dependence of the enthalpy change might also be explained by weak Ca^2+^ binding to EF2, since the other five Ca^2+^ coordinating ligands remain intact in this mutant. The identity of the Ca^2+^ binding sites was explored further using kinetic methods.

Previously, we found that Ca^2+^ dissociates from S100A4 with a rate constant of 20 s^− 1^, as determined by the change in intrinsic tyrosine fluorescence.^18^ S100A4 contains two tyrosine residues (one in each of the EF-hand domains), and mutating each in turn to phenylalanine enabled us to identify the source of the signal change. Both Y19F and Y75F constructs showed tyrosine fluorescence changes on Ca^2+^ dissociation with amplitudes about half that of the wild type (Fig. S1c). However, the rate constants were increased (Y19F, 113 s^− 1^; Y75F, 45 s^− 1^), suggesting some loss of stability of the Ca^2^^+^-bound form with the mutants. We also examined the E33Q and D63N mutants under similar conditions and found that the D63N mutant gave no resolved transient, suggesting that Ca^2+^ binding was weak and/or its release was too fast to measure (> 400 s^− 1^). On the other hand, the E33Q mutant gave a transient that was similar in amplitude to the wild type but with double the rate constant (Fig. S1d; 49 s^− 1^ cf. 22 s^− 1^), indicative of a small loss in the stability of the EF2 Ca^2+^ binding loop. The data are consistent with the ITC data in that they indicate that the D63N mutation leads to a marked weakening of Ca^2+^ binding and suggests that Ca^2+^ dissociation from EF2 is responsible for tyrosine fluorescence change. On the other hand, tyrosine residues in both the EF2 domain (Y75) and the EF1 domain (Y19) respond to the loss of Ca^2+^ from the EF2 site (Fig. S1a), thus demonstrating communication between the two EF hands.

**Fig. 3:**
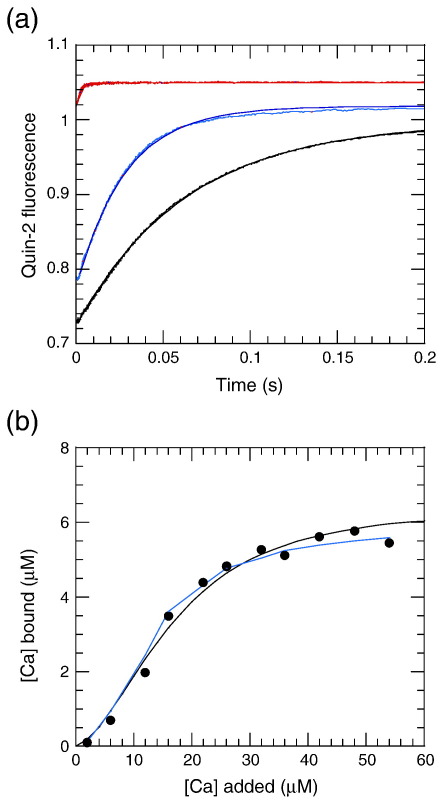
(a) Dissociation of Ca^2+^ from wild-type S100A4 (black), E33Q mutant (blue), and D63N mutant (red), as monitored by Quin-2 fluorescence. Fits to a single exponential yielded rate constants of 16 s^− 1^, 35 s^− 1^, and 500 s^− 1^, respectively, but the D63N record had a small amplitude, suggesting that most of the signal change was too fast to measure. The records for the mutants were vertically offset for clarity. (b) Ca^2+^ binding to S100A4 as determined from the amplitude of the resolved transient (*k* ≈ 20 s^− 1^) when 6.5 μM S100A4 was preincubated with various added [Ca^2+^] and then mixed with 100 μM Quin-2. The amplitude was converted into a bound concentration from an independent calibration using the same Quin-2 solution and known free [Ca^2+^]. The latter transients were too fast to measure but gave a steady signal on the seconds timescale. The continuous black line was computed for a model with cooperative binding in which the first Ca^2+^ bound to EF2 of an S100A4 dimer with a *K*_d_ of 100 μM and the second Ca^2+^ bound to EF2 of an S100A4 dimer with a *K*_d_ of 1.7 μM. The blue line was computed for a model with an EF1 binding site with *K*_d_ = 0.25 μM that was optically silent in the Quin-2 assay (i.e., dissociation too fast to measure) and an EF2 site with *K*_d_ = 5 μM. While either model could account for the data, the measured stoichiometry from ITC (Table 1) favors the cooperative model.

In order to increase the signal-to-noise ratio of the Ca^2+^ dissociation transients, we used Quin-2 as combined fluorescent indicator and high-affinity chelator.^19,20^ These measurements confirmed that Ca^2+^ dissociated from wild-type protein with a rate constant of 16 s^− 1^ (Fig. 3a; cf. 20 s^− 1^ for the tyrosine signal). Given the poor signal-to-noise ratio of the tyrosine signal, the difference is barely significant but may reflect the fact that the Quin-2 signal represents at least a two-step sequential reaction (Ca^2+^ dissociation from S100AS4, followed by rapid binding to Quin-2). The E33Q mutant showed a similar amplitude but with a slightly higher rate constant (35 s^− 1^), indicating that the EF2 Ca^2+^ binding site remained functional despite a compromised EF1 site. The D63N mutant showed a much smaller transient that was just resolved (500 s^− 1^) and could represent Ca^2+^ release from the compromised EF2 site with a 30-fold reduction in affinity, as suggested by the ITC data. Ca^2+^ binding to EF1 therefore appears weaker than Ca^2+^ binding to EF2, and its dissociation is too fast to measure by stopped-flow methods in all constructs.

Some S100A4 preparations showed a second slow phase of Ca^2+^ dissociation (generally < 20% of the signal) with a rate constant of 0.4–4 s^− 1^. The amplitude of the slow phase increased on storage of S100A4 at 4 °C and probably arose from oligomerisation and/or oxidation. This factor is important to control for in Ca^2+^ dissociation measurements in the presence of target peptides (described below), where the observed rate constants are comparable to this slow phase.

The amplitude of Quin-2 records contains information on the amount of Ca^2+^ bound. In a separate experiment, the Quin-2 fluorescence signal obtained in the stopped-flow apparatus was calibrated by the addition of known Ca^2+^ concentrations, and the baseline fluorescence in the absence of contaminating Ca^2+^ (+ 1 mM EGTA) was determined. These data indicated that the resolved signal corresponded to one Ca^2+^ bound per S100A4 monomer (Fig. 3b), again suggesting that EF2 alone is responsible for this transient.

### Myosin peptide binding to S100A4

**Scheme 1:**
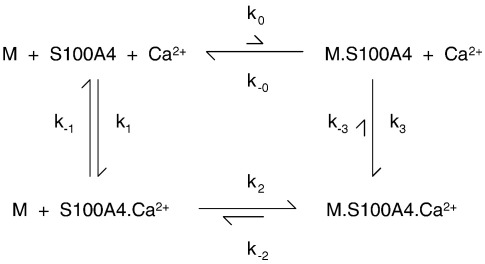
Ca2+ and myosin peptide (M) binding to S100A4.

The interaction of myosin tail fragments (M) with S100A4 depends on [Ca^2+^], with little detectable binding in the absence of Ca^2+^.^2,7,17^ Scheme 1 presents a minimal pathway describing the thermodynamics and kinetics of this interaction, where arrow lengths indicate the approximate position of the equilibria involved.

**Fig. 4:**
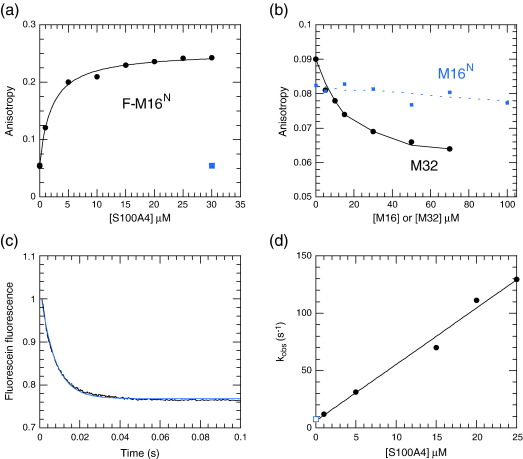
(a) The binding of fluorescein-labelled M16^N^ myosin peptide (0.1 μM) to S100A4 based on fluorescence anisotropy. Titration (circles) was carried out in 20 mM NaCl, 10 mM Hepes, 1 mM Mg^2+^, and 100 μM Ca^2+^ at pH 7.5 and 20 °C. The fitted hyperbola yielded an apparent *K*_d_ of 2.0 ± 0.23 μM (see the text for correction). At the end of the titration, EGTA was added to 1 mM, causing the peptide to dissociate (square datum). (b) Displacement of F-M16^N^ (5 μM) from S100A4 (5 μM) by increasing [M32] (circles) or [M16^N^] (squares). The continuous line corresponds to a *K*_d_ of 3 μM for M32 binding to S100A4, as determined by a fit to a competitive single binding site equation, using the Excel solver routine to minimise the variance. The continuous line deviated systematically from the data when the modelled *K*_d_ for M32 was increased or reduced by 50% of its value. M16^N^ caused little displacement of its fluorescent counterpart even at a concentration of 200 μM. Modelling to a competitive binding equation indicates that the *K*_d_ of M16^N^ for S100A4 is ≥ 100 μM (broken line). (c) Stopped-flow record of 0.1 μM F-M16^N^ binding to 25 μM S100A4 monitored by fluorescein emission intensity. A fit to a single exponential yields *k*_obs_ = 138 s^− 1^. (d) Dependence of the pseudo first-order rate constant *k*_obs_ for F-M16^N^ (0.1 μM) binding as a function of S100A4 concentration. The rate constant of 7.7 s^− 1^ (open square) at zero [S100A4] was obtained by the displacement of FM16^N^ from S100A4 by excess M32 (see Fig. S2a). The fitted straight line yields *k*_on_ = 4.9 μM^− 1^ s^− 1^ and *k*_off_ = 6.2 s^− 1^. At concentrations above 25 μM S100A4, the graph became nonlinear and reached a maximum *k*_obs_ of 150 s^− 1^.

Myosin peptides were used to investigate the minimal S100A4 target site on the myosin heavy chain. We confirmed that the fluorescein-tagged peptide F-M16^N^ bound to S100A4 using a fluorescence anisotropy assay^16^ and yielded an apparent *K*_2_ of 2.0 μM (assuming a 1:1 binding between each S100A4 monomer and the peptide) in the presence of Ca^2+^ (Fig. 4a). This value is very close to that reported by Malashkevich *et al.*, who found a *K*_2_ of 0.89 μM in 20 mM KCl when [S100A4] was defined in terms of dimer concentration.^17^ However, in our titration, we noted about a 35% quench in the total fluorescein fluorescence intensity (*I*_vv_ + 2*I*_vh_; see Materials and Methods) on binding, which means that the observed anisotropy is weighted towards the unbound state. Consequently, the true *K*_d_ is overestimated by about this percentage (i.e., our corrected *K*_2_ = 1.3 μM with respect to monomer concentration; Table 1). More importantly, we found that the unlabelled M16^N^ peptide is a very poor competitor of F-M16^N^ and binds with a *K*_2_ of ≥ 100 μM (Fig. 4b). A larger peptide, M32, however binds with a *K*_2_ of around 3 μM, as determined by competition with F-M16^N^ binding. In view of the discrepancy with the results of Malashkevich *et al.* in the length of the “minimal” peptide, we checked the sequence of our commercial M16^N^ sample and found it to be correct.^17^ We also checked for M16^N^ binding to S100A4 using other assays (see the text below) and confirmed that it was at least an order of magnitude weaker than M32 binding. In the absence of Ca^2+^, F-M16^N^ binding was undetectable (< 10% change in anisotropy), indicating that *K*_0_ is > 300 μM (Fig. 4a). We therefore conclude that the fluorescein moiety contributes to much of the binding energy of F-M16^N^ on interaction with S100A4 in the presence of Ca^2+^, and that the “minimal” binding peptide encompasses at least the region 1907–1938 (≡ M32) of myosin heavy chain IIA.

The hypothesis that the fluorescein of F-M16^N^ interacts directly with S100A4 was also supported by the change in its fluorescence emission intensity on binding, indicative of a changed environment of the fluorescein group. This change was exploited to determine the kinetics of F-M16^N^ binding to S100A4 by stopped-flow methods (Fig. 4c). The observed pseudo first-order rate constant for binding (*k*_obs_ = *k*_2_[S100A4] + *k*_−_ _2_) as a function of [F-M16^N^] yielded association (*k*_2_) and dissociation (*k*_−_ _2_) rate constants of 4.9 μM^− 1^ s^− 1^ and 6.2 s^− 1^, respectively (Fig. 4d). The dissociation rate constant was confirmed by measuring the rate constant for the displacement of F-M16^N^ with a large excess of M32 (Fig. S2a). These rate constants yield a *K*_2_ of 1.3 μM, identical with the corrected value from the equilibrium anisotropy assay (Table 2). When a solution of 0.1 μM F-M16^N^ and 10 μM S100A4 in 50 μM Ca^2+^ was mixed with 1 mM EGTA, a rise in fluorescence was noted with a *k*_obs_ of 9.4 s^− 1^ (Fig. S2b). We assign this rate constant to the dissociation of Ca^2+^ from the F-M16^N^–S100A4–Ca^2+^ complex (*k*_−_ _3_), with subsequent rapid dissociation of F-M16^N^ via *k*_−_ _0_, because the S100A4 concentration (> *K*_2_ = 1.3 μM) would ensure that the flux through the other route (via *k*_− 2_ and *k*_− 1_ in Scheme 1) would be low (the calculated *k*_obs_ through this route: *k*_obs_ = 16 × 1.3/(1.3 + 10) = 1.8 s^− 1^). From the overall thermodynamic balance of Scheme 1, the estimated rate constants lead to a *k*_3_ value of > 5 × 10^8^ M^− 1^ s^− 1^, which is close to the diffusion-controlled limit, for Ca^2+^ binding to the preformed F-M16^N^–S100A4 complex. The kinetics of M32 binding, as far as determined, were comparable to those of F-M16^N^ (see Supplementary Data).

### Effect of peptides and myosin fragments on S100A4–Ca^2+^ dissociation kinetics

**Fig. 5:**
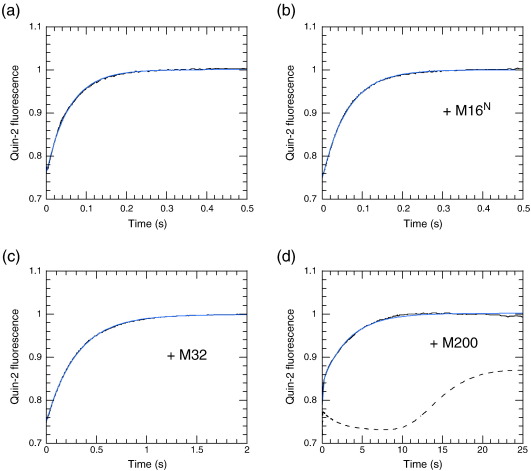
Dissociation of Ca^2+^ from S100A4 in the absence and in the presence of target peptides monitored using Quin-2 fluorescence. (a) Mixed with 100 μM Quin-2 was 10 μM S100A4 + 50 μM Ca^2+^ (stopped-flow reaction chamber concentrations). A superposed fit to a single exponential yields *k*_obs_ = 17 s^− 1^. (b) As in (a), but with 50 μM M16^N^ premixed with S100A4 (*k*_obs_ = 16 s^− 1^). (c) As in (a), but with 50 μM M32 premixed with S100A4 (*k*_obs_ = 3.3 s^− 1^). (d) As in (a), but with 2.5 μM M200 premixed with 5 μM S100A4. A biphasic exponential fit yielded rate constants of 13 s^− 1^ (*A* = 26%) and 0.30 s^− 1^ (*A* = 74%). The broken line shows increased light scatter at 336 nm as the released M200 formed filaments in the absence of Ca^2+^ with a 10-s lag and indicates that this process did not influence the profile of Quin-2 fluorescence. Experiments were carried out in either 20 mM NaCl, 20 mM Tris, 1 mM DTT, and 100 μM Ca^2+^ at pH 7.5 and 20 °C (a and b; cf. Malashkevich *et al.*^17^), or 100 mM NaCl, 10 mM Hepes, and 100 μM Ca^2+^ at pH 7.5 and 20 °C (c and d). Similar profiles were observed for experiments (a and b) performed in the latter buffer and yielded rate constants of 16 s^− 1^, indicating a weak binding of M16^N^ under both conditions. Rate constants were unaffected by the inclusion of 1 mM Mg^2+^.

Ca^2+^ dissociation measurements for wild-type S100A4, using Quin-2 as described above, were repeated as a control for the effect of target peptides on this process. On mixing 10 μM S100A4 in the presence of Ca^2+^ with 100 μM Quin-2, the observed profile was fitted to a single exponential with *k*_obs_ = 17 s^− 1^ (Fig. 5a; cf. *k*_− 1_ in Scheme 1). The observed Ca^2+^ dissociation rate constant from S100A4 was unaffected by the presence of 50 μM M16^N^ (i.e., the peptide without fluorescein label) when premixed with S100A4 (Fig. 5b), whereas the same concentration of M32 caused the rate constant to be reduced to 3.3 s^− 1^ (Fig. 5c). This observation supports the above findings that M32 binds much more tightly to S100A4 than does M16^N^. The concentration dependence of the observed Ca^2+^ dissociation profile confirmed that M32 bound to S100A4 with a *K*_d_ in the micromolar region (*K*_2_; Scheme 1). When the stopped-flow traces were fitted to a single exponential, the observed rate constant *k*_obs_ as a function of [M32] could be fitted to a quadratic equation to yield a limiting rate constant of 3.2 s^− 1^ at saturating [M32], compared with 17 s^− 1^ in the absence of M32 (Fig. S3a). The former represents the dissociation rate constant of Ca^2+^ from the M32–S100A4–Ca^2+^ complex (*k*_−_ _3_; Scheme 1). Such an analysis assumes that the M32 binding kinetics are fast relative to the Ca^2+^ dissociation kinetics. The alternative model that can be solved analytically is that for slow M32 binding. In the latter case, the stopped-flow traces at intermediate [M32] would be biphasic, with the relative amplitude of the slow phase (*k*_−_ _3_ = 3.2 s^− 1^) increasing at the expense of the fast phase (*k*_− 1_ = 17 s^− 1^) with increasing [M32]. A comparison of fits to a single exponential and fits to a biphasic exponential with fixed rate constants and variable amplitudes (i.e., both equations had three free parameters) to the data obtained at 0.25 μM M32 showed small deviations in the residuals with similar magnitudes but opposite signs. This suggests that the kinetics of Ca^2+^ and M32 dissociation from the M32–S100A4–Ca^2+^ complex are comparable (*k*_−_ _2_ ≈ *k*_−_ _3_), as might be expected from the kinetics of competition with F-M16^N^ binding (Supplementary Data). However, while both fast binding models and slow binding models are approximations, they yielded similar values for the apparent *K*_d_ for M32 binding (*K*_2_ = 0.4 μM), which is smaller than that deduced by the anisotropy competition assay, and also suggested a stoichiometry closer to 0.5 mol of M32 per S100A4 monomer than 1:1.

Similar measurements were made to investigate the dissociation of Ca^2+^ from S100A4 in the presence of the coiled-coil fragments M111 and M200, revealing a slower release of Ca^2+^ (0.3–0.5 s^− 1^; Fig. 5d; Fig. S3b). When increasing concentrations of M111 were added to S100A4, the rate constants of the fast and slow phases remained approximately constant (20 s^− 1^ and 0.4 s^− 1^), while the amplitudes interchanged. This is indicative of a slow equilibrium between free M111 and bound M111 relative to the rate constant for Ca^2+^ release. In the case of M200, the measurement was complicated by the fact that, in 100 mM NaCl, this fragment forms filamentous aggregates (see the text below). However, on addition of twice the stoichiometric amount of S100A4 in the presence of Ca^2+^, the aggregates were solubilised and the solution had low turbidity. On mixing with excess Quin-2, free Ca^2+^ was rapidly chelated, and the M200–S100A4–Ca^2+^ complex dissociated. The liberated M200 then formed filaments. Simultaneous measurement of Quin-2 fluorescence and light scatter showed that the latter reaction had a substantial lag and that the Ca^2+^ dissociation phase was essentially complete before the aggregation phase began (Fig. 5d). Consequently, there was little optical interference from the change in light scatter on the measured Ca^2+^ dissociation kinetics. Furthermore, similar Ca^2+^ dissociation kinetics were obtained in 200 mM NaCl, where M200 remains soluble (Fig. S5a). However, as Quin-2 chelates Ca^2+^, the increasing absorbance of Quin-2 gave rise to a small inner filter effect that initially reduced the observed light scatter signal. The major phase of the Quin-2 signal had a rate constant (*k*_− 3_) of 0.3 s^− 1^ attributed to Ca^2+^ dissociation from the M200–S100A4–Ca^2+^ complex (Fig. 5d), a value similar to that for the M111 complex. It is likely that once Ca^2+^ dissociates, the M200–S100A4 complex also rapidly dissociates (*k*_−_ _0_ ≫ *k*_−_ _3_), but the increase in light scatter is not observed immediately because filament formation has a slow nucleation event (see the text below). The slower dissociation of Ca^2+^ from the M111 and M200 complexes (0.3 s^− 1^), compared with the M32 complex (3.2 s^− 1^) and the F-M16^N^ complex (9.4 s^− 1^), could be a consequence of the stabilisation of a multimeric complex due to the coiled-coil nature of the fragments and/or because myosin residues beyond the minimal binding region identified above (residues 1907–1938 ≡ M32) are involved (see Discussion).

The binding of Ca^2+^ and a target peptide to S100A4, as shown in Scheme 1, is thermodynamically coupled so that *K*_1_*K*_2_ = *K*_0_*K*_3_. For simplicity, we assume a 1:1 stoichiometry and independent binding sites, which are unlikely to be true at least for the longer fragments. However, the trends in binding remain valid for more complex cases. Binding of target peptides to S100A4 in the absence of Ca^2+^ is weak (*K*_0_ > 100 μM), while binding of target peptides to S100A4 in the presence of Ca^2+^ (*K*_2_) is around a micromolar or less for longer fragments. Knowing that Ca^2+^ binding to S100A4 has *K*_1_ = 2–5 μM (from the ITC data above and the literature^14,17,18^) requires that Ca^2+^ binding to the M–S100A4 complex be very tight (*K*_3_ < 0.15 μM and even lower for M200). The latter cannot be easily determined directly as it requires protein concentrations greater than *K*_0_ (i.e., >>100 μM) so that the proteins remain bound together throughout the titration with Ca^2+^. However, *K*_3_ is readily determined by calculation because values or limits for *K*_0_, *K*_1_, and *K*_2_ are available (Table 2). This aspect is addressed further below for M200, which has the complication of forming filaments at the ionic strength used.

### NMR spectroscopy of peptide complexes

**Fig. 6:**
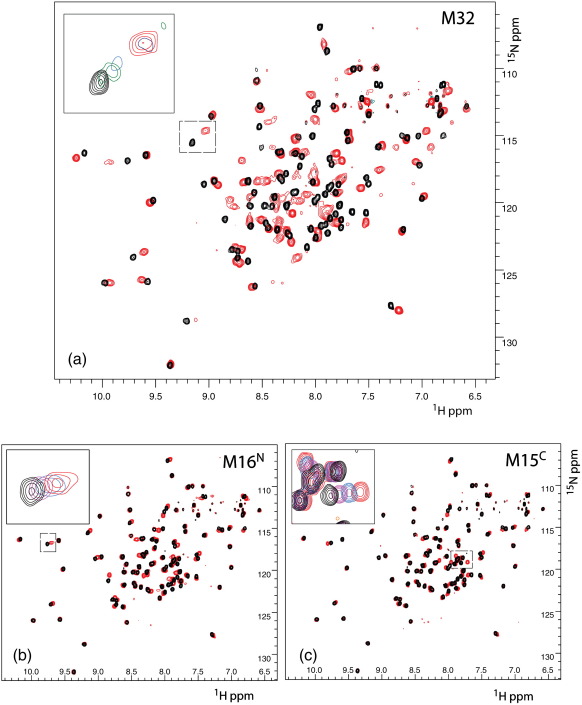
Chemical shift changes in the 600-MHz ^1^H,^15^N HSQC spectra of 0.2 mM ^15^N-labelled Δ10-S100A4 on addition of myosin peptides at 35 °C. (a) Superposition of the spectra in free form (black) and in the presence of a 3-fold molar excess of M32 (red). The inset shows progressive changes in the spectra on peptide addition (intermediate exchange) corresponding to the following protein/peptide molar ratios (left to right): free (black), 1:0.25 (green), 1:0.5 (light blue), 1:1.5 (dark blue), and 1:3 (red). Cross-peaks corresponding to 1:0.75 and 1:1 molar ratios are too broad to be observed. (b) Superposition of the spectra in free form (black) and in the presence of a 4-fold molar excess of M16^N^ (red). The inset shows progressive changes in the spectra on peptide addition (fast exchange) corresponding to the following protein/peptide molar ratios (left to right): free (black), 1:1 (magenta), 1:2 (light blue), and 1:4 1:3 (red). (c) Superposition of the spectra in free form (black) and in the presence of a 4-fold molar excess of M15^C^ (red). The inset shows progressive changes in the spectra on peptide addition (fast exchange) corresponding to the following protein/peptide molar ratios (left to right): free (black), 1:1 (magenta), 1:2 (light blue), and 1:4 1:3 (red).

The contributions of different myosin regions to the interaction with S100A4 were analysed by NMR. To observe the direct perturbations of protein resonance on binding, we used truncated Δ10-S100A4, which lacks 10 C-terminal residues. From our data^18^ and reported data,^17^ these residues in crystal form bind to the active site of the protein and can stabilise the tetrameric state at the high protein concentrations used for NMR studies. However, these residues are not involved in any contact in the dimeric form of the protein. The removal of the C-terminus had a minor effect on the NMR spectra, with the ^1^H,^15^N heteronuclear single-quantum coherence (HSQC) spectrum exhibiting highly dispersed cross-peaks of uniform intensity that correspond to a stable globular fold (Fig. 6). No interaction was observed for Δ10-S100A4 in the absence of Ca^2+^, as expected from the above kinetic studies. In the presence of Ca^2+^, the largest perturbations in the NMR spectra were detected on the addition of M32 (Fig. 6a). In the course of the titrations, the majority of resonances displayed chemical shift changes accompanied by severe broadening for many signals, leading to a complete disappearance for some of them (Fig. 6a, inset). At M32 concentrations above equimolar, the resonance broadening was gradually reduced; at a Δ10-S100A4/M32 ratio of 1:3, most of the HSQC cross-peaks can be detected, although a significant number of cross-peaks still remained more broadened than the rest. These spectral changes correspond to the intermediate-exchange regime between the free state and the bound state on Δ10-S100A4. The exchange rate for M32 binding can be estimated from the chemical shift difference between the free state and the bound state for the cross-peak highlighted in Fig. 6a (inset). This cross-peak displays extensive broadening accompanying a progressive chemical shift change characteristic of the intermediate exchange, with the chemical shift difference (rad s^− 1^) comparable to the exchange rate constant *k*_ex_ = *k*_on_ + *k*_off_. The shift difference of 0.12 ppm at 600 MHz gives the exchange rate constant estimate of *k*_ex_ ∼ 450 s^− 1^ at 35 °C, in fair agreement with the *k*_2_[S100A4] + *k*_− 2_ value determined by stopped-flow analysis at 20 °C in Table 2. A similar intermediate-exchange regime had been previously characterised for the binding of S100A11 to its target annexin A2.^28^

In contrast, the titration of Δ10-S100A4 with either M16^N^ or M15^C^ demonstrated fast exchange between the free state and the bound state (Fig. 6b and c, insets). In both cases, no significant broadening was detected at the intermediate peptide concentrations, and the range of chemical shift changes at equivalent peptide concentrations was much smaller than for M32. The chemical shift changes on the addition of M16^N^ were similar to the reported effect of the corresponding peptide^17^ and were larger than on the addition of M15^C^. The lower range of chemical shift changes and the faster exchange between the free state and the bound state for M16^N^ and M15^C^, in comparison to M32, demonstrate that neither of the smaller peptides possesses the binding affinity of the longer M32 fragment. While the addition of M16^N^ affects more resonances and the range of chemical shift changes is larger than for M15^C^, the latter contributes significantly to the binding, as evidenced by the specific chemical shift changes and the increase of affinity for M32 that span both regions. The affinities of M16^N^ and M15^C^ were too low to be measured by NMR, as the binding site was far from saturation even at a 9-fold molar excess of the peptides at 0.2 mM Δ10-S100A4.

**Fig. 7:**
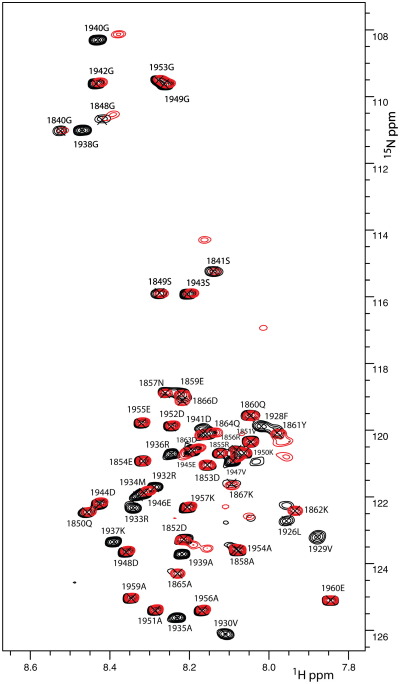
Chemical shift changes in the ^1^H,^15^N HSQC spectra of ^13^C,^15^N-labelled M111 on addition of full-length S100A4. Superposition of the spectra in free form (black) and in the presence of a 1.5 molar excess of S100A4 (red). Resonance assignments derived from the analysis of triple-resonance spectra are marked. In addition to sharp intense cross-peaks, the spectra contain broad low-intensity peaks that cannot be assigned due to low sensitivity.

The involvement of the region corresponding to M15^C^ can be directly detected by NMR using larger recombinant myosin fragments. We used full-length S100A4 for this analysis because the effect of the C-terminus on the interaction with this tight-binding myosin fragment was negligible. The ^1^H,^15^N HSQC spectrum of M111 (residues 1850–1960; Fig. 1) shows a number of sharp intense peaks (Fig. 7). The majority of the sharp resonances correspond to the C-terminal region 1926–1960 at the end of the coiled-coil, agreeing with the prediction that this region is unstructured and highly dynamic. The rest of the sharp signals were assigned to the N-terminal 1850–1866 region, demonstrating that it is also unstructured. No resonances corresponding to the coiled-coil region 1866–1925 were detected in the triple-resonance experiments, although a number of low-intensity broad cross-peaks are present in the ^1^H,^15^N HSQC spectra in addition to the signals from the unstructured parts of the protein. The formation of coiled coil in the 1866–1925 region results in a highly elongated structure with a slow rotational diffusion rate, leading to relaxation broadening of the NMR resonances. This correlates with the experimental NMR observation showing that the M111 fragment is long enough to form a stable coiled-coil structure. The formation of the coiled coil correlates with the high helical content detected for the fragment by CD below 20 °C (Fig. S4b) and electron microscopy (see the text below).

The addition of full-length S100A4 leads to the additional broadening of resonances in the 1926–1939 region and chemical shift changes for the resonances corresponding to G1940 and D1941 (Fig. 7), while the resonances corresponding to the 1942–1960 region are unaffected. The selective broadening of myosin resonances demonstrates that the 1926–1939 fragment is immobilised in the complex and is involved in a direct interaction with S100A4. This fully correlates with the enhanced binding of the M32 fragment, compared to the M16^N^ peptide, and the chemical shift perturbations of the S100A4 resonances on addition of the M15^C^ peptide that incorporates the 1926–1939 region. In agreement with the stopped-flow experiments, the NMR data show that the 1926–1939 fragment provides an essential contribution to the S100A4–myosin interaction.

### Electron microscopy

**Fig. 8:**
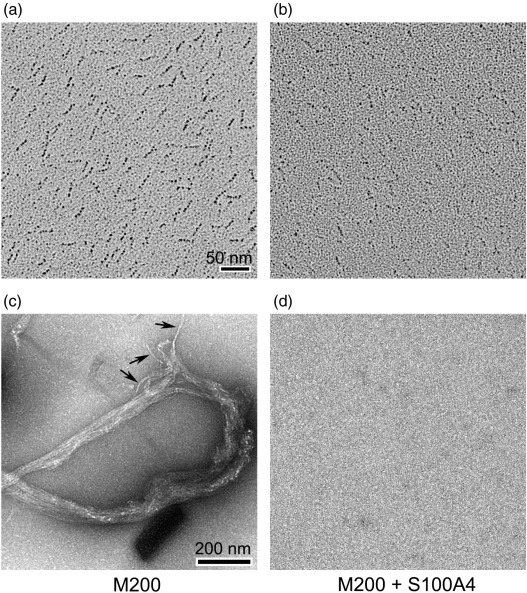
Characterisation of myosin IIA coiled-coil tail fragments. Metal-shadowed electron micrographs of (a) M200 and (b) M111 deposited from 0.5 M ammonium acetate. Scale bar represents 50 nm. (c and d) Electron micrographs of a negatively stained M200 tail fragment. Fields of M200 (c) in the absence and (d) in the presence of a 2-fold molar excess of S100A4, respectively. Arrows point to individual strands emerging from images of filamentous bundles. The samples were prepared in 0.1 M sodium acetate buffer, where M200 formed large filamentous aggregates but were solubilised in the presence of S100A4. Scale bar represents 200 nm (see Materials and Methods for details).

Rotary shadowed images of M200 and M111 deposited from high-ionic-strength solutions (0.5 M ammonium acetate) confirmed that these myosin fragments formed coiled-coil structures (Fig. 8a and b). The mean length of M200 was 26.4 ± 2.03 nm (mean ± SD; *n* = 132), consistent with about 170 residues forming a stable coiled coil and 30 residues comprising the nonhelical C terminus, which were not visualised by electron microscopy (Fig. S4a). Using a value of 0.1485 nm per residue for a coiled coil^29^ yields an expected length of 25.2 nm. The M111 preparation appeared more heterogeneous, probably because the helical structure is only marginally stable at 20 °C, as determined by circular dichroism (Fig. S4b).

In 0.1 M Na-acetate, M200 formed filamentous bundles (Fig. 8c) that were almost 100 nm wide and several micrometers long. Seen within the structures are individual strands about 10 nm wide (Fig. 8c, arrows), a width comparable to that formed by intact NM-MHC IIA.^30^ From the dimensions of the individual M200 coiled-coil structures (26 nm × 2 nm), it is evident that the aggregates may contain in excess of 10,000 molecules; in this respect, they are poor models of the native nonmuscle myosin IIA filament (400 nm long and containing 28 molecules^30^). However, the relatively open structure of the filament bundle allows their rapid solubilisation on addition of S100A4. On addition of a two times stoichiometric concentration (i.e., 2× S100A4 dimer per M200 coiled-coil fragment) in the presence of Ca^2+^, no trace of filamentous bundles was observed (Fig. 8d). These results were observed both when the soluble M200 sample was diluted into a 0.1 M Na-acetate solution containing S100A4 and when S100A4 was added to preformed M200 filaments and incubated for 15 min.

### Kinetics of M200 filament formation and solubilisation

M200 filament formation is also evident from the increased turbidity of the solution, measured as an apparent absorbance at 300 nm. The turbidity shows a sharp transition between 100 mM and 200 mM NaCl, with a midpoint value of about 120 mM NaCl when 5 μM M200 was used (Fig. S5a). Over the range of 0–1 absorbance, the turbidity of M200 in 100 mM NaCl buffer was practically linear with concentration (Fig. S5b). The small offset on the *x*-axis indicated a critical concentration of ∼ 0.1 ± 0.04 μM M200 at this ionic strength. The value is difficult to determine accurately from this experiment, but it is clear that if M200 shows a well-defined critical concentration, then its value is < 0.2 μM. If a line is fitted just to the data obtained at [M200] between 2.5 μM and 5 μM, the intercept is 0.49 μM, indicating there is a slight upswing in the turbidity with concentration. It is possible that, at these concentrations, the turbidity increases more due to aggregation of existing smaller filaments.

**Fig. 9:**
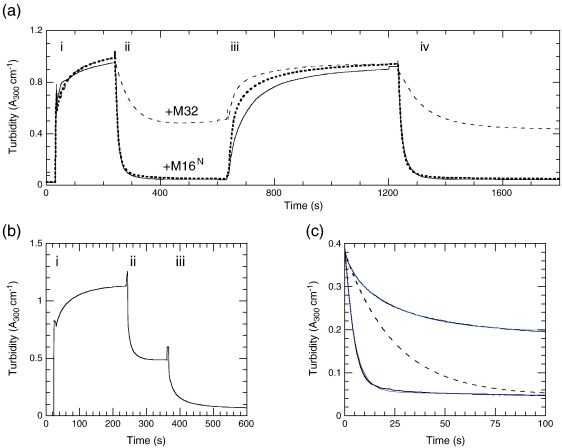
Dissociation of M200 filaments by S100A4. (a) Initially, M200 was added (i) from a concentrated stock in 0.5 M NaCl to give a final concentration of 5 μM M200 (with respect to the polypeptide chain) in 100 mM NaCl, 10 mM Hepes, and 0.1 mM Ca^2+^ at pH 7.5 and 20 °C. A biphasic rise in turbidity was recorded for 200 s (continuous line). Then 10 μM S100A4 (with respect to monomer concentration) was added (ii), followed by 0.2 mM EGTA (iii) and 1 mM Ca^2+^ (iv). The experiment was repeated in the presence of 250 μM M32 (broken line) or 250 μM M16^N^ (dotted line). (b) M200 was diluted (i) into 100 mM NaCl to form filaments as in (a), then 5 μM S100A4 was added (ii), causing a 54% drop in turbidity. A further 5 μM S100A4 (iii) caused turbidity to fall to almost zero. (c) Stopped-flow records monitoring the dissociation of 5 μM M200 filaments on jumping [NaCl] from 100 mM to 120 mM NaCl. Other components of the buffer were 20 mM Hepes, 1 mM Mg^2+^, and 100 μM Ca^2+^ at pH 7.5 and 20 °C. The upper trace, obtained in the absence of S100A4, showed a 45% decrease in turbidity with rate constants of 0.33 s^− 1^ (22% amplitude) and 0.037 s^− 1^ (78% amplitude). In the presence of 20 μM S100A4 (lower trace), the turbidity decreased by 87% with a rate constant of 0.2 s^− 1^. The broken line shows the calculated profile when the disassembly of the M200 filaments occurred only by sequestration of monomeric M200 by S100A4 with a rate constant of 0.04 s^− 1^, which gives the same initial rate as observed in the absence of S100A4 (see the text).

Turbidity assays provide a convenient way to monitor the kinetics of filament solubilisation by S100A4.^31^ We found that a 2-fold molar excess of S100A4 monomer per M200 polypeptide chain was required to fully solubilise the M200 filament aggregate in the presence of Ca^2+^ (Fig. 9a and b). Addition of excess EGTA reversed the process, allowing M200 filaments to reform, while addition of further Ca^2+^ caused resolubilisation (Fig. 9a). Both filament assembly and disassembly appeared at least biphasic processes, with the first phase being resolvable just by manual mixing. Stopped-flow studies confirmed that the reactions did indeed occur on the seconds timescale and that manual mixing was sufficient to record most of the profiles satisfactorily (Fig. S6a and b). However, on removal of Ca^2+^ with EGTA, there was a lag of a few seconds before M200 filament formation (Fig. S6b) (as was noted above when Quin-2 was used as chelator) (Fig. 5d), which was not resolved in manual mixing assays.

**Scheme 2:**
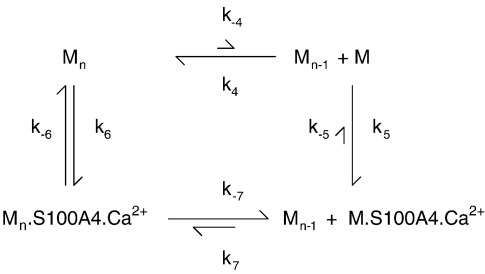
M200 filament (Mn) dissociation by S100A4.

The kinetics of the polymerisation and depolymerisation of M200 are likely to be complex, as M200 monomers could add to the ends or the sides of filaments, and the filaments themselves may bundle, fragment, or anneal. For the purposes of discussion, we will consider the dissociation of one M200 monomer (M) from a filament bundle (denoted M_*n*_), as shown in Scheme 2.

It is of interest to know whether S100A4 drives depolymerisation solely by binding to free M monomer and allowing the polymer equilibrium to readjust (i.e., via *k*_−_ _4_ and *k*_5_), or whether S100A4 also binds to the filament bundle and actively depolymerises an M200 unit (via *k*_6_ and *k*_−_ _7_). It is known that S100A4 can bind to myosin rod fragments,^8,17^ but this in itself does not prove that there is significant flux via *k*_−_ _7_. In an attempt to address this question, the dissociation rate of the M200 filament was followed on jumping the NaCl concentration from 100 mM to 120 mM in the absence and in the presence of S100A4 (Fig. 9c). Under these conditions in the absence of S100A4, turbidity dropped to about 50% of its initial value with a profile fitted to a double exponential (*k*_1obs_ = 0.33 s^− 1^ with 12% amplitude and *k*_2__obs_ = 0.037 s^− 1^ with 43% amplitude, relative to the total amplitude expected for a full dissociation). Therefore, at the end point of this reaction, the filament concentration decreased by about 2-fold (turbidity is proportional to concentration over this range; Fig. S5b). There should also be a corresponding rise in M200 monomer concentration, but this may be less than accounted for by the loss of filaments if the drop in turbidity was also associated with a rise in small M200 oligomers with a lower specific turbidity. The underlying kinetics for this reaction is complex, with the exponential-like character having possible contributions from: (i) the depletion of the initial filament concentration (i.e., a first-order-like reaction) and a buildup of monomers and small oligomers that could repolymerise; (ii) zero-order kinetics (i.e., linear) for dissociation restricted to filament ends but with length-dependent dissociation rate constants (i.e., progressively smaller rate constants as the filament shortens), as proposed for synthetic skeletal myosin filaments;^32^ or (iii) a nonlinear relationship between specific turbidity and filament size^10^ so that the initial fragmentation of larger filaments contributes to more signal change than the disassembly of small oligomers at the latter stages of the reaction. The practically linear relationship observed in Fig. S5b argues that (iii) is unlikely to be the dominant contribution. However, regardless of the detailed mechanism, if S100A4 dissociated M200 filaments simply by sequestering the free M200 monomers, then it is likely that the initial rate of the depolymerisation reaction, when monomer reassociation is negligible, would be unaffected by S100A4 and that only the amplitude would change. This can be modelled empirically with an exponential function having a 100% amplitude and a rate constant of 0.04 s^− 1^ (Fig. 9c, broken line) to give the same initial rate as a reversible reaction with a 12% amplitude and an observed rate constant of 0.33 s^− 1^ (i.e., modelling *k*_obs_ = *k*_off_ + *k*_on_ and amplitude = 100 × *k*_off_/(*k*_off_ + *k*_on_)%). In the presence of 20 μM S100A4 (the concentration after the NaCl jump), the reaction had almost double the amplitude (indicating near-full dissociation of the M200 filament) and was closer to a single exponential (*k*_obs_ = 0.19 s^− 1^; a biphasic fit yielded a 85% amplitude with *k*_obs_ = 0.21 s^− 1^). Significantly, the initial rate (i.e., slope) was five times faster than in the absence of S100A4 (Fig. 9c). This indicates that S100A4 bound to the M200 filament aggregate and caused an enhanced rate of monomer dissociation. A similar conclusion was reached from measurements made for a 2-fold dilution at 100 mM NaCl (data not shown). However, here, the turbidity change on dilution in the absence of S100A4 was only 10% of the amplitude obtained in the presence of S100A4; thus, the kinetics of the former were dominated by the association rate constant.

Further information on Scheme 2 was obtained from competition assays. In the presence of M32, the solubilisation of M200 was incomplete owing to competition for the S100A4; however, relatively high concentrations of M32 were needed to give a detectable effect (Fig. 9a, broken line). In the presence of 5 μM M200, 250 μM M32 was required to give a 50% inhibition in the amplitude of turbidity change. These data yield an estimate of *K*_5_ ≈ 0.001 μM, which represents the binding of S100A4–Ca^2+^ to free M200. An independent estimate of *K*_5_ based on the Ca^2+^ dependence of the turbidity assay yielded a value of 0.003 μM (see Supplementary Data). Thus, monomeric M200 (i.e., coiled-coil dimer) binds 3 orders of magnitude tighter than M32 to S100A4. The presence of 250 μM M16^N^ had no effect on the amplitude of the turbidity changes in M200 (Fig. 9a, dotted line), confirming that it binds more than an order of magnitude weaker to S100A4 than M32.

**Fig. 10:**
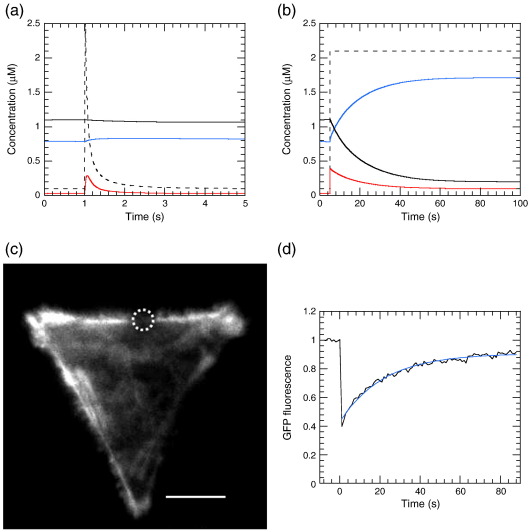
(a) Simulated response of the kinetic pathway (Scheme 1) to a single wave of Ca^2+^ (broken line) with a rise time constant of 10 ms and a decay constant of 100 ms. For simplicity, the myosin filament was modelled as a cooperative system 5M ↔ M_5_ (see Fig. S7 for further details). The total concentrations of the components were 2 μM S100A4 and 2 μM myosin (monomer concentration). Filament (black line), S100A4–Ca^2+^ (red line), and M–S100A4–Ca^2+^ (blue line). (b) As in (a), but with the response to a step increase in free Ca^2+^ to 2 μM. (c) FRAP of GFP–myosin IIA expressed in an A431/SIP1 cell immobilised on a Y-fibronectin pattern (CYTOO Chip™). The TIRF image is derived from a sequence 1 s after the bleaching of a 3-μm-diameter spot (broken circle) across a stress fibre. Scale bar represents 10 μm. (d) FRAP time course of the bleached spot in (c), normalised against an unbleached region of the filament, to yield a recovery rate constant of 0.046 s^− 1^.

Knowledge of the equilibrium and rate constants for Ca^2+^ binding to free S100A4 and target complexes is of value in estimating the degree of interaction of these proteins in cells, where the resting cytoplasmic [Ca^2+^] is around 0.1 μM and rises transiently to 1–10 μM on excitation. Using the rate and equilibrium constants determined for M200 (Scheme 1, Table 2) and a simplified model for filament formation (see Supplementary Data), we calculated the time courses for a single Ca^2+^ pulse (Fig. 10a) and a step increase in free Ca^2+^ (Fig. 10b). As expected from the data in Fig. S6c, at the initial resting Ca^2+^ of 0.1 μM, there is significant interaction between S100A4 and myosin when both are present at 2 μM concentration, such that 40% of the total myosin is present as the soluble M–S100A4–Ca^2+^ complex (Fig. 10a, blue line). In the absence of S100A4, only 6% of the myosin is present in monomeric form (the parameters for filament formation were chosen to give a free myosin concentration close to the estimated critical concentration of 0.1 μM). However, a brief Ca^2+^ pulse has little further effect on the concentration of filamentous myosin, as its kinetics are too slow to respond (Fig. 10a, black line). On the other hand, S100A4 does interact with Ca^2+^ on this timescale, but the concentration of S100A4–Ca^2+^ (Fig. 10a, red line) remains small owing to its relatively weak binding (modelled with *K*_d_ = 4 μM). For a significant decrease in the concentration of myosin filaments (Fig. 10b, continuous black line), the Ca^2+^ concentration needs to remain above 1 μM for tens of seconds. A train of pulses over this timescale would also be effective in partially dissociating the myosin filaments to an extent dependent on the average Ca^2+^ concentration (Fig. S7).

The above modelling assumes that the kinetics of M200 filament disassembly measured *in vitro* (Fig. 9c) occurs on a timescale similar to that of myosin filaments *in vivo*. To test for this, we performed fluorescence recovery after photobleaching (FRAP) measurements on A431 cells expressing green fluorescent protein (GFP)–myosin IIA (Fig. 10c). In the absence of activating transcription factors, these cells do not express significant amounts of S100A4 and in the absence of epidermal growth factor, the mean cytoplasmic [Ca^2+^] is close to resting levels. The cells were immobilised on Y-shaped fibronectin patterns to give a reproducible arrangement of stress fibres spanning the apices of the cell.^33^ Photobleaching recovery occurred over a 3-μm diameter with a time constant of around 20 s (Fig. 10d). While analysis of FRAP kinetics is complex, it appears to be dominated by the dissociation rate constant of GFP–myosin from the stress fibre, rather than diffusion, as the stress fibre intensity recovered with practically the same time course along its 3-μm length.^34^ Furthermore, monomeric myosin in the cytoplasm would be expected to diffuse across the 3-μm diameter within a few seconds. These studies confirm that for a Ca^2+^-dependent control of myosin filament dissociation via a calcium-binding protein such as S100A4, the mean free [Ca^2+^] would need to remain elevated for tens to hundreds of seconds. However, even at resting levels of Ca^2+^, S100A4 expressed at micromolar levels in the cytoplasm should be effective in dissociating myosin filaments.

## Discussion

In this article, we reexamine aspects of Ca^2+^ and NM-MHC IIA tail fragment binding to S100A4 and extend the study through new kinetic measurements. Ca^2+^ binds to the S100A4 dimer with an affinity of around 2–6 μM. ITC measurements indicate a stoichiometry of one Ca^2+^ per S100A4 monomer and a *K*_d_ of 1.7 μM, with a second site with *K*_d_ = 6 μM revealed at temperatures of ≥ 30 °C. Regarding the assignment of the sites to EF1 or EF2, the E33Q mutant appears more similar to wild-type S100A4 than the D63N mutant and has an intact EF2 site, as determined by tyrosine and Quin-2 fluorescence (Fig. 3a; Fig. S1b). The weaker binding site of native S100A4 seen by ITC (Fig. 2a) would then correspond to EF1, as assumed by Malashkevich *et al.*^17^ The resolved Quin-2 signal therefore reflects Ca^2+^ that was bound to EF2, with Ca^2+^ release from EF1 being too fast to measure. In the presence of target peptides, the amplitude of the Quin-2 signals increases by no more than 20%, suggesting that the release of any Ca^2+^ bound to EF1 remains too fast to measure. The small increase in amplitude of the resolved phase could reflect the increase in the initial degree of binding to EF2 at a fixed free [Ca^2+^], in line with the decrease in *K*_d_ for Ca^2+^. Previously, we reported a stoichiometry of binding of 1.7 ± 0.18 Ca^2+^ bound per S100A4 monomer, based on the amplitude of the tyrosine fluorescence change on addition of Ca^2+^.^18^ These signals were much noisier than those recorded using the Quin-2 indicator reported here. Furthermore, the calculated stoichiometry is critically dependent on the accuracy of the S100A4 concentration determination. S100A4 lacks tryptophan, and the absorbance from the two tyrosine residues is weak, so the conclusions are dependent on the accurate correction for turbidity and the lack of significant UV-absorbing contaminants. However, the unchanged amplitude of the Quin-2 signal on Ca^2+^ dissociation from the wild type compared with the E33Q mutant (Fig. 3a) argues strongly that a single site (EF2) per monomer is involved. Nevertheless, there is communication between the EF1 domain and the EF2 domain on dissociation of Ca^2+^ from EF2, as indicated by tyrosine fluorescence (Fig. S1c), which could account for the NMR findings of Dutta *et al.*, who concluded that EF1 had a higher affinity.^15^

We confirm that a 16-amino-acid myosin peptide derivative, F-M16^N^, binds to S100A4 in the presence of Ca^2+^ with an affinity of around 1 μM,^17^ but we consider the fluorescein moiety to make a large contribution to the binding energy because the corresponding unlabelled M16^N^ peptide binds 2 orders of magnitude more weakly. The data of Malashkevich *et al.* imply that the unlabelled M16^N^ peptide binds almost as tightly as longer fragments, although no raw data are shown for this peptide.^17^ In our analysis (Fig. 4b), M32 and F-M16^N^ were assumed to bind to each monomer independently. However, the stoichiometry of M32 binding (Fig. S3a) suggests that one M32 peptide may span two monomers of S100A4, so it could compete with two molecules of F-M16^N^. Fitting to such a model yields a *K*_d_ of 1.5 μM for the M32 peptide (i.e., half that of the simple model). From our measurements, a 32-mer peptide (corresponding to A1907-G1938 of the myosin sequence) represents a minimal binding region. Longer fragments (M111 and M200) bind with even higher affinity, indicating that additional amino acids of the myosin heavy chain contribute to the binding interaction and/or the capacity of the longer fragments to form coiled-coil structures enables higher-order structures to form with increased avidity. We are currently exploring longer peptides to address this question and consider that both factors are involved.

Based on the S100A4 concentration determined from the absorbance at 280 nm, one mol of M32 peptide binds to two S100A4 monomers. In support of this conclusion, the ITC titration curve of an R1893-R1923 myosin fragment of Malashkevich *et al.* (their Fig. 11^17^) shows a stoichiometry close to 0.5 mol of peptide per mole of S100A4 (dimer), in which concentrations of the components were determined from quantitative amino acid analysis. The site between helix III and helix IV of S100A4, exposed in the presence of Ca^2+^, could accommodate an α-helical target of about 16 residues. The longer M32 minimal peptide identified above suggests that the myosin binding site may extend beyond a single S100A4 monomer, as was reported for the novel interactions of a 31-mer SIP (*S*iah-*i*nteracting *p*rotein) fragment with the S100A6 dimer, as determined by NMR.^35^ The conclusion regarding the S100A4: peptide target stoichiometry reported here requires confirmation by comparable structural methods.

A corresponding stoichiometry is also seen in the interaction of S100A4 with M200, where 2 mol of S100A4 monomer was required to solubilise 1 mol of M200 aggregate (when expressed as M200 polypeptide chain concentration) (Fig. 9b). Murakami *et al.*^31^ found that a S100A4/M200 ratio of 5:1 was required for solubilisation in their assays, whereas Li *et al.*^11^ found that 1 mol of S100A4 dimer was required to disassemble 1 mol of NM-MHC IIA rods. These stoichiometries complicate the calculation of the equilibrium parameters for the models described in Schemes 1 and 2. If the binding sites remained independent, then *K*_d_ values would be altered by just a statistical factor but would more likely extend the potential for cooperativity. However, until accurate stoichiometries are determined by direct structural methods, Schemes 1 and 2 provide a useful start point for the discussion of thermodynamic coupling. As a consequence of this coupling in Scheme 1, the very weak binding of target proteins to S100A4 in the absence of Ca^2+^ requires the target-bound form of S100A4 to bind Ca^2+^ with a very high affinity (Table 2). In the case of bound F-M16^N^, the estimated *K*_d_ limit for Ca^2+^ binding is < 20 nM. It is important to stress that this would not be the observed *K*_d_ for Ca^2+^ binding to a mixture of F-M16^N^ and S100A4, unless the peptide concentration was much greater than millimolar (i.e., exceeded *K*_0_ in Scheme 1), because the observed constant would reflect both the binding of peptide to S100A4 and the binding of Ca^2+^ to this complex. A similar argument applies to M200, but here the thermodynamic coupling factor appears even greater (possibly because of avidity due to the two potential binding sites from each chain of the coiled coil of M200). The physiological consequence of this is that the myosin tail and S100A4 could bind at resting cytoplasmic Ca^2+^ levels and cause filament disassembly, provided at least one of the protein components is present at a concentration exceeding several micromolars (cf. Fig. S6c). Unfortunately, there are no reliable estimates of the effective concentrations of S100A4 in cells, although total concentrations of micromolar might be expected.^22^ Cell excitation that leads to elevated [Ca^2+^] would promote further myosin filament disassembly. However, the modelling shown in Fig. 10 indicates that a sustained rise in [Ca^2+^] lasting tens of seconds is required in order to achieve a significant effect.

In the absence of added Ca^2+^ (i.e., where the free “contaminant” [Ca^2+^] in buffers would be of the order of 1 μM), mixing S100A4 with excess Quin-2 produced a small-amplitude signal (corresponding to 0.1 mol of Ca^2+^ per mole of S100A4 monomer) with a rate constant of around 1 s^− 1^. This is similar to the rate constants obtained for target-bound S100A4 and suggests that this process might correspond to the dissociation of Ca^2+^ from a small fraction of the S100A4 tetramers. The crystal structure of the Ca^2^^+^-bound form of the S100A4 tetramer shows that the C termini of two of the monomers are positioned in the target binding sites of two opposing subunits.^18^ Indeed, in this article, we drew attention to the C-terminal sequence ExFPxxxP, which is similar to the DLPFVVP sequence identified within the minimal myosin target peptide (Fig. 1). An S100A4 construct lacking the last 13 C-terminal residues fails to from higher oligomers beyond the dimer.^17^ The tetramer and higher oligomers of the wild type are therefore unlikely to bind to the myosin target site with significant affinity because of the competition with the C-terminus. Based on thermodynamic coupling, the tetramer would also be expected to have a higher affinity for Ca^2+^ than for the dimer. Indeed, ultracentrifuge data^17^ suggest that the affinity would be about 20-fold higher. A Ca^2+^ *K*_d_ value of around 0.1 μM for the tetramer would suggest that overexpression of S100A4 could lead to oligomerisation of S100A4 at resting cytoplasmic [Ca^2+^]. In addition, phenothiazine drugs have been reported to induce S100A4 oligomers and to inhibit interaction with NM-MHC IIA.^36^ It is possible that there are natural effectors that operate by this mechanism.

S100A4 solubilises M200 filament aggregates in the presence of Ca^2+^; in terms of kinetics, this construct serves as a model system for studying the mechanism of depolymerisation of intact NM-MHC IIA filaments. S100A4 binds to M200 filament aggregates and actively depolymerises them, rather than just binding to the monomer and perturbing the myosin filament equilibrium. However, the (M200)_*n.*_S100A4.Ca^2+^ filament complex is only a transient species that leads to a solubilised M200.S100A4.Ca^2+^ complex. This has implications for *in vivo* studies, since the degree of colocalisation of S100A4 with myosin filaments may be rather limited under steady-state conditions, and any solubilisation would lead to a more general cytoplasmic distribution of both proteins. Zhang *et al.* found that an eCFP-S100A4 construct did interact with a NM-MHC IIA–eYFP rod construct in the cytoplasm of HeLa cells, using fluorescence lifetime imaging.^37^ As these experiments required 5 min of acquisition time, the time-averaged Ca^2+^ concentration was likely to be close to resting levels and thus corroborates the potential for these proteins to interact at Ca^2+^ concentrations significantly lower than the *K*_d_ for S100A4 in the absence of target. However, the *in vivo* case is complicated by further regulatory mechanisms such as myosin phosphorylation^38^ and cross-reactivity between other S100 proteins and their targets.

## Materials and Methods

### Peptides and proteins

M32, M16^N^, and M15^C^ peptides (Fig. 1) with an acetylated N-terminus and an amidated C-terminus at 95% purity were purchased from Peptide 2.0, Inc. (Chantilly, VA). The composition of the peptides was confirmed by mass spectroscopy, and the sequence of the M16^N^ fragment was also confirmed by tandem mass spectrometry fragmentation and cyanogen bromide cleavage. F-M16^N^, a fluorescein derivative linked to M16^N^ via an aminohexanoic acid linker, was obtained from Biosynthesis, Inc. (Lewisville, TX) and was identical with FITC-MIIA^1908–1923^ as used by Malashkevich *et al.*^17^

The mutant forms of S100A4 were made using the QuikChange Site-Directed Mutagenesis kit (Stratagene) and the following oligonucleotides:

5′ CCACCTTCCACAAGTTCTCGGGCAAAGAGG 3′

and 5′ CCTCTTTGCCCGAGAACTTGTGGAAGGTGG 3′ for Y19F S100A4;

5′ GGACTTCCAAGAGTTCTGTGTCTTCCTGTC 3′

and 5′ GACAGGAAGACACAGAACTCTTGGAAGTCC 3′ for Y75F S100A4;

5′ GCTCAACAAGTCACAGCTAAAGGAGCTGC 3′

and 5′ GCAGCTCCTTTAGCTGTGACTTGTTGAGC 3′ for E33Q S100A4; and

5′ GATGAGCAACTTGAACAGCAACAGGGAC 3′

and 5′ GTCCCTGTTGCTGTTCAAGTTGCTCATC 3′ for D63N S100A4. To check that no spurious changes had arisen as a result of the mutagenesis reactions, we resequenced the entire S100A4 gene in each mutant construct.

Recombinant wild-type and mutant forms of S100A4 were expressed and purified from *Escherichia coli* BL21(DE3) cells. Transformed cells were grown overnight at 37 °C in 2× YT media supplemented with 100 μg mL^− 1^ ampicillin. The overnight culture (15 mL) was used to inoculate 1 L of 2× YT media containing 100 μg mL^− 1^ ampicillin. The cultures were grown at 37 °C until the OD_600_ had reached a value of ∼ 0.8, and then isopropyl 1-thio-β-d-galactopyranoside (to a final concentration of 0.25 mM) was added. The temperature was reduced to 30 °C, and incubation continued overnight. Cells were harvested by centrifugation (20 min, 6000 rpm, 4 °C), and cell pellets were resuspended in lysis buffer [50 mM NaH_2_PO_4_, 300 mM NaCl, and 10 mM imidazole (pH 8.0)] supplemented with two Complete Protease Inhibitor Tablets (Roche). Cells were lysed by sonication (6 × 30 s pulses with 30-s intervals using an MSE Soniprep 150 sonicator). After sonication, DNase I and MgCl_2_ (to a final concentration of 20 mM) were added, and the suspension was stirred for 30 min at 4 °C. The lysate was centrifuged at 18,000 rpm for 50 min, and the cell-free extract was loaded onto a 20-mL column of Ni-NTA Superflow resin (Qiagen) equilibrated in lysis buffer. The resin was washed with 400 mL of wash buffer [50 mM NaH_2_PO_4_, 300 mM NaCl, and 20 mM imidazole (pH 8.0)], and the protein was eluted using a linear gradient ranging from 20 mM to 250 mM imidazole in 50 mM NaH_2_PO_4_ and 300 mM NaCl (pH 8.0). S100A4-containing fractions were pooled and concentrated using a centrifugal 3000 molecular weight cutoff filter unit (Millipore). The protein was further purified using a Superdex 75 gel-filtration column equilibrated in 10 mM Hepes, 20 mM NaCl, and 2 mM DTT (pH 7.5) buffer. Pure S100A4-containing fractions eluted from the column were frozen in aliquots and stored at − 80 °C. Expression and purification of M111 and M200 were similar to those for S100A4, except that the buffer used for the Ni-NTA column contained 500 mM NaCl for M200 and the final gel-filtration column (and subsequent protein storage) contained 100 mM NaCl (for M111) or 500 mM NaCl (for M200), respectively, and lacked DTT. Uniformly ^15^N-labelled S100A4 was expressed in 2× M9 minimal medium using 4 g L^− 1^ glucose and 1 g L^− 1 15^NH_4_Cl. Uniformly ^15^C,^15^N-labelled M111 was expressed in 2× M9 minimal medium using 2 g L^− 1^ [^13^C]glucose and 1 g L^− 1 15^NH_4_Cl. Labelled proteins were purified following the same protocols as for unlabelled proteins.

### Isothermal titration calorimetry

ITC data were collected using a VP-ITC microcalorimeter (MicroCal Ltd., Northampton, MA) and analysed by fitting to a single-site binding equation or a two-site binding equation using MicroCal Origin software.

### Absorption and turbidity measurements

Absorption spectra were recorded using a Cary 50 spectrophotometer (Varian Ltd., Walton on Thames, UK). Protein concentrations were calculated from their absorbance at 280 nm, using molar coefficients of 2980 M^− 1^ cm^− 1^ (M200 and S100A4) and 1490 M^− 1^ cm^− 1^ (M111), based on their tyrosine content (two residues per chain and one residue per chain, respectively). Myosin fragments were measured in 0.5 M NaCl buffer, and care was taken to correct for the small contribution from turbidity. Protein concentrations are stated in terms of their monomeric polypeptide chain concentration. Under the conditions used for kinetic analysis, S100A4 is predominantly a dimer,^21,25^ and M200 and M111 form stable coiled coils; therefore, the molar protein concentrations are half those given in all three cases. Fluorescence emission spectra were also checked, and those preparations containing a detectable shoulder at 340 nm due to contaminating tryptophan-containing proteins were subjected to further purification. F-M16^N^ concentrations were based on fluorescein absorbance (ɛ_490_ = 78,000 M^− 1^ cm^− 1^). Other peptide concentrations (M32 and M16: > 95% purity) were based on their dry weight concentration. Quin-2 concentrations were calculated from ɛ_354_ = 5000 M^− 1^ cm^− 1^ in the absence of Ca^2+^.^39^ Turbidity assays used to monitor M200 filament formation and solubilisation with manual mixing were carried out using a 10-mm pathlength and a 0.1-ml microcuvette (105.250-QS; Hellma, Southend on Sea, UK) at a wavelength of 300 nm. For determination of the free Ca^2+^ concentration, Fura-2 fluorescence excitation spectra were measured in the same cuvette using the 2-mm path for excitation to minimise inner filter effects.

CD spectra were measured on a JASCO J-715 spectropolarimeter equipped with a JASCO PTC-348WI temperature-controlled unit at 0.05 mM protein solutions using a 1-mm pathlength microcuvette.

### Fluorescence spectroscopy

Fluorescence spectra and anisotropy measurements were recorded using an SLM 8000 spectrofluorimeter (SLM, Urbana, IL). Fluorescein anisotropy measurements were carried out in L-format using a 515-nm emission filter. The four combinations of vertical and horizontal polarisers (vv, vh, hh, and hv) were each recorded for 30 s, and then the initial setting (vv) was re-read to check for signal stability. Calculations of the total fluorescence (*I*_vv_ + 2*I*_vh_) and anisotropy (*I*_vv_ − *I*_vh_/(*I*_vv_ + 2*I*_vh_)), corrected for the *g* factor (hh/hv), were performed using an Excel spreadsheet. Competitive equilibrium binding data were analysed by an iterative fit to a single-site model using the Excel Solver routine (Microsoft) in which the *K*_d_ for M32 was a free parameter, while that for F-M16^N^ was fixed at 2 μM. The latter was determined by a fit to a hyperbola to an independent titration in the absence of M32 (Fig. 2a) under conditions where [F-M16^N^] = 0.1 μM ≪ *K*_d_). Note that the small correction for the emission intensity weighting to the observed anisotropy was not applied to these competitive titrations as the error will tend to cancel.

### Stopped-flow spectroscopy

Stopped-flow measurements were carried out using an SX18-MV apparatus (Applied Photophysics Ltd., Leatherhead, UK) with a dead time of 1.5 ms.^40^ Records were generally captured on a logarithmic time base. Absorption (turbidity) measurements were usually recorded using a 10-mm pathlength, while fluorescence was recorded with a 2-mm pathlength for excitation and emission. The stated concentrations of components refer to the reaction chamber after mixing, unless otherwise indicated. Tyrosine fluorescence was excited at 280 nm, and the emission was collected through a UG11 bandpass filter plus a 305-nm longpass filter.^18^ Fluorescein fluorescence was excited at 480 nm, and the emission was detected through a 515-nm longpass filter. Quin-2 fluorescence was measured by excitation at 336 nm, and emission was collected with a 455-nm longpass filter. For simultaneous recording of light scatter, a second channel was monitored through a UG11 filter. Rate constants were obtained by fitting to single-exponential or double-exponential functions using Kaleidagraph (Synergy Software) or Applied Photophysics Pro-Data software. Modeling of kinetic schemes was performed using Berkeley Madonna software.

### Fluorescence microscopy

A clone of human epidermoid carcinoma A431/SIP1 cells,^41^ with constitutive expression of the GFP-tagged heavy chain of nonmuscle myosin IIA, was used for FRAP experiments. To generate the clone, we cotransfected A431/SIP1 cells with pGFP-NMMII-A^42^ and pPuro, as described previously.^41^ GFP-MHC-IIA expression was confirmed by Western blot analysis with antibodies to GFP (AMS Biotechnology, UK) and MHC-IIA (Covance, Princeton, NJ). A431-SIP1 cells, stably expressing GFP–nonmuscle myosin IIA, were seeded on medium Y-shaped micropatterns (CYTOO SA, Grenoble, France) following the manufacturer's protocol and allowed to adhere for 3 h.

FRAP was carried out using a custom-built total internal reflection fluorescence (TIRF) apparatus based on a Zeiss 135 TV Axiovert microscope.^43^ Excitation light (488 nm, 15 mW) from an argon-ion laser (model 35LAP43; Melles Griot, Huntingdon UK), after beam expansion to 6 mm diameter, was divided equally into two beams using a nonpolarising prism beamsplitter. One beam was used to image the GFP-labelled cell using prism-based TIRF, while the second bleaching beam entered the back of the objective lens (63× 1.2 NA Zeiss C-apochromat water immersion) via the standard excitation path and was brought to a point focus near the centre of the field. The photobleaching beam had an independent aperture and electronic shutter to control the profile of the bleaching beam and the extent of bleaching. The specimen was imaged using an iXon DV887 emCCD camera (Andor Technology, UK) and translated at the manual stage until the region of interest was in the predefined position of the bleaching beam. The bleaching beam was generally operated for 1–2 s, and images were acquired at 1-s intervals thereafter.

### NMR spectroscopy

All NMR samples were prepared in 20 mM 4-morpholineethanesulfonic acid, 20 mM NaCl, 5 mM CaCl_2_, and 5% D_2_O buffer (pH 6.1). DTT (2 mM) was added to the samples containing S100A4. Titration experiments were carried out at 0.2 mM S100A4 using 10 mM peptide solutions. Spectra for M111 backbone resonance assignments were measured using 0.4 mM protein solution. NMR spectra were obtained at 298 K using Bruker AVANCE DRX 600 or AVANCE DRX 800 spectrometers, which are both equipped with cryoprobes. Proton chemical shifts were referenced to external 2,2-dimethyl-2-silapentane-5-sulfonate. The ^15^N and ^13^C chemical shifts were referenced indirectly using recommended gyromagnetic ratios.^44^ Spectra were processed with TopSpin (Bruker) and analysed using Analysis.^45^ Three-dimensional HNCO, HN(CA)CO, HNCA, HN(CO)CA, HNCACB, and HN(CO)CACB experiments were used for the sequential assignment of the backbone NH, N, CO, C^α^, and C^β^ resonances.

### Electron microscopy

For negative staining, the stock solution of the M200 tail fragment was diluted with a solution consisting of 0.1 M Na-acetate, 3 mM MgCl_2_, 0.1 mM CaCl_2_, 10 mM imidazole, and 5 mM NaH_2_PO_4_ (pH 7.0), with or without 14 μM S100A4, to give a final concentration of 7 μM M200. Following dilution, 5 μl of the mixture was applied to a grid with a thin carbon film supported by a holey carbon film, and the grid was negatively stained using 1% uranyl acetate. For metal shadowing, the stock solutions of the M200 and M111 tail fragments were diluted with 0.5 M ammonium acetate solution to give a final concentration of 5 μM protein. This was mixed with an equal volume of glycerol, and the resulting mixture was sprayed onto freshly cleaved mica then rotary shadowed with platinum at an angle of 6°.^46^ Grids were examined in a Philips CM120 electron microscope (FEI, Hillsboro, OR) operated at 80 kV. Images were recorded on a 2000 × 2000 F224HD slow-scan CCD camera (TVIPS, Gauting, Germany) at a magnification of 65,000× (0.37 nm pixel^− 1^). The lengths of nonoverlapping well-shadowed molecules were measured as described by Burgess *et al.*^47^

## Figures and Tables

**Table 1:** Analysis of ITC data for Ca^2+^ binding to S100A4

S100A4	*n*_1_ (Ca^2+^ per monomer)	*K*_1_K_1d_ (μM)	Δ*H*_1_ (cal mol^− 1^)	*N*_2_ (Ca^2+^ per monomer)	*K*_2_K_2d_ (μM)	Δ*H*_2_ (cal mol^− 1^)
Wild type, 30 °C	0.83 ± 0.2	1.7 (+ 1.7/− 0.6)	+ 1224 ± 216	0.42 ± 0.27	5.7 ± 1.1	− 1907 ± 2210^a^
Wild type, 10 °C	0.95 ± 0.014	1.6 ± 0.4 (cooperative)	− 930 ± 20	—	—	—
E33Q, 30 °C	0.33 ± 0.03	10.9 ± 1.9	+ 1643 ± 162	—	—	—
E33Q, 10 °C	0.96 ± 0.09	∼ 1 cooperative	< − 83	—	—	—
D63N, 30 °C	0.54 ± 0.01	68 ± 1	+ 4136 ± 43	—	—	—
D63N, 10 °C	0.44 ± 0.01	65 ± 3	+ 2584 ± 62	—	—	—

a Standard error exceeds estimate. Therefore, this parameter remains ill-defined. Data were obtained as described in Fig. 2.

**Table 2:** Summary of rate and equilibrium constants defined according to Scheme 1

Myosin peptide/fragment	*K*_0_ (μM)	*K*_1_ (μM)	*k*_1_k_1_ (μM^− 1^ s^− 1^)	*k*_−_ _1_ (s^− 1^)	*K*_2_ (μM)	*k*_2_ (μM^− 1^ s^− 1^)	*k*_−_ _2_ (s^− 1^)	*K*_3_ (μM)	*k*_3_ (μM^− 1^ s^− 1^)	*k*_−_ _3_ (s^− 1^)
F-M16^N^	> 300	5	3.2	16	1.3	4.9	6.2	< 0.02	> 500	9.4
M16^N^	ND	Above values common to all targets			> 100	ND	ND			
M32	> 100				3	∼ 2	∼ 6	< 0.15	> 16	3.2
M200	> 100				∼ 0.003	ND	< 0.3	< 0.00015	> 2000	0.3

ND, not determined. The limit of *K*_3_ was calculated from *K*_3_=*K*_1_*K*_2_/*K*_0_.
