# Supplementary material for: Mechanism of the Ca2+-Dependent Interaction between S100A4 and Tail Fragments of Nonmuscle Myosin Heavy Chain IIA
Source: J Mol Biol. 2011 Jan 28;405(4-3):1004–26. doi: 10.1016/j.jmb.2010.11.036 (PMC3025356; doi:10.1016/j.jmb.2010.11.036)
Supplement: Supplementary file 1 — Supplementary materials [file mmc1.pdf]

Supplementary Text.

Kinetics of M32 binding to S100A4

In order to asses the kinetics of M32 binding, a competition assay was set up in which an equimolar concentration of F-M16^N and M32 was mixed with equimolar S100A4. The decrease in fluorescence, associated with F-M16^N binding was about half the amplitude observed in the absence of M32, indicating that their K_d's were comparable. However, the fluorescence quench showed a 12% overshoot, suggesting that F-M16^N binding was slightly faster. Modelling the profile indicated that the association rate constant, k_2, for F-M16^N binding was about twice as large as that of M32, while their dissociation rate constants were similar.

The equilibrium constant (K_5) for dissociation of the M200.S100A4.Ca^{2+} complex

The value of K_5 (scheme 2) was estimated from two independent experiments. From the competition between M32 and M200 binding (Fig. 9a), it was found that 250 μM M32 was required to give a 50% inhibition in the amplitude of the turbidity change arising from 5 μM M200 at saturating Ca^{2+} concentrations. Under these conditions the free S100A4.Ca^{2+} concentration is ~ 0.06 μM based on $K_2 = 3$ μM = [S100A4.Ca^{2+}][M32]/[M32.S100A4.Ca^{2+}] and knowing that at the end point of the solubilisation phase about half the S100A4.Ca^{2+} (5 μM) is bound to M32 and half is bound to M200. The free M200 concentration will remain close to its critical concentration ($K_4 = 0.1$ μM), so that $K_5 = 0.1 * 0.06 / 5 \approx 0.001$ μM, which represents the binding of S100A4.Ca^{2+} to free M200.

A second estimate was calculated from the apparent Ca^{2+} binding constant observed in the turbidity assay (Fig. S6c). 5 μM M200 in the presence of S100A4 and 1 mM EGTA gave a high turbidity in 0.1 M NaCl buffer, as S100A4 binds weakly under these conditions. Addition of aliquots of Ca^{2+} caused filament dissociation as the free Ca^{2+} rose, to give an apparent binding constant for Ca^{2+} of 0.14 μM (Fig. S6c). At

the mid-point of this titration, the M200 filament concentration = 2.5 μM (with respect to M200 polypeptide content), and therefore the concentration of M200.S100A4.Ca^{2+} ≈ 2.4 μM. (assuming the free M200 remains near its critical concentration (0.1 μM) throughout). The remaining S100A4 (2.6 μM) is either free S100A4 (2.5 μM) or S100A4.Ca^{2+} (0.07 μM) in a ratio defined by the free Ca^{2+} (0.14 μM) and K_2 = 5 μM (Table 2). From these estimates, the binding of M200 monomer to S100A4.Ca^{2+} defined by K_5 = 0.1 * 0.07/2.4 ≈ 0.003 μM, which is the same order of magnitude as deduced from competition with M32.

Fig. S1.

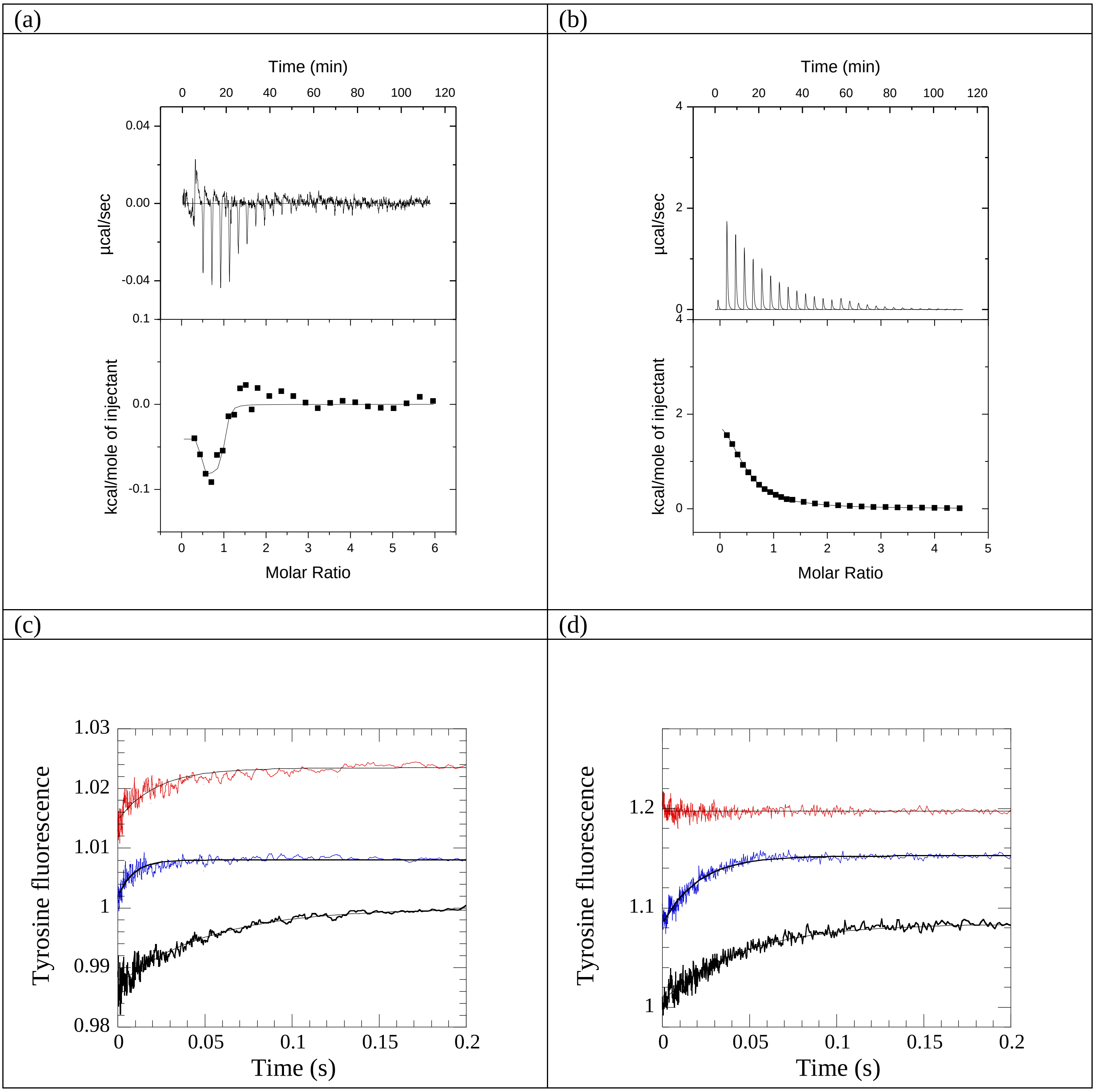

Fig. S1. Isothermal titration calorimetry records for Ca^{2+} binding to S100A4 constructs (a) 75 μM E33Q construct at 10 ^{o}C, (b) 300 μM D63N construct at 10 ^{o}C. The conditions were as in Fig. 2 of main text. (c)

Dissociation of Ca^{2+} from wild type S100A4 (black), Y19F mutant (blue) and Y75F mutant (red) monitored by tyrosine fluorescence. 13 μM of each S100A4 construct plus 100 μM Ca^{2+} was mixed with 200 μM EGTA (reaction chamber concentrations) and the tyrosine fluorescence monitored. The buffer was 20 mM NaCl, 10 mM HEPES, 2 mM DTT at pH 7.5 and 20 ^{o}C. Fits to a single exponential function yielded rate constants of 20 s^{-1}, 113 s^{-1} and 45 s^{-1} for wild-type, Y19F mutant and Y75F mutant respectively. (d) Dissociation of Ca^{2+} from wild type S100A4 (black), E33Q mutant (blue) and D63N mutant (red) monitored by tyrosine fluorescence. 13 μM of each S100A4 construct plus 15 μM Ca^{2+} was mixed with 200 μM EGTA (reaction chamber concentrations) and the tyrosine fluorescence monitored. Fits to a single exponential function yielded rate constants of 22 s^{-1} and 49 s^{-1} for wild-type and E33Q construct respectively, while that for D63N was too fast to measure. The records for the mutants were vertically offset for clarity in both figures.

Fig. S2.

(a) (b)

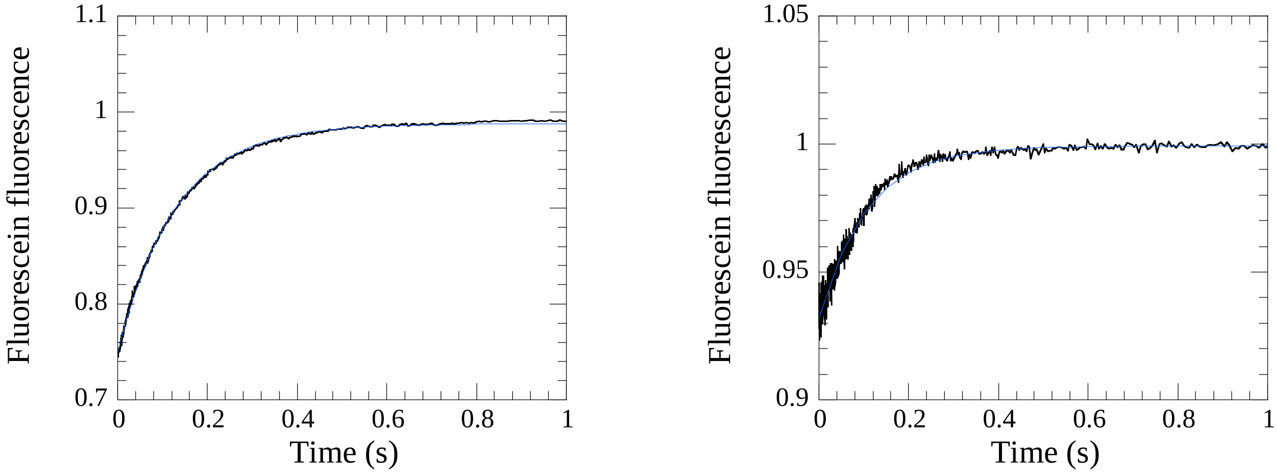

Fig. S2. (a) Displacement of 0.1 μM F-M16^N from 5 μM S100A4 by 60 μM M32 in the presence of Ca^{2+} monitored by fluorescein emission intensity. A single exponential fit yielded a rate constant of 7.7 s^{-1}. (b) Dissociation of Ca^{2+} from the F-M16^N.S100A4.Ca^{2+} complex. 0.1 μM F-M16^N +10 μM S100A4 + 50 μM Ca^{2+} was mixed with 1 mM EGTA. A single exponential fit yielded a rate constant of 9.3 s^{-1}. Note in these stopped-flow measurements of fluorescein intensity, no polarising optics were present at the magic angle and therefore the observed intensity change may have a contribution from a change in anisotropy.

Fig. S3.

(a) (b)

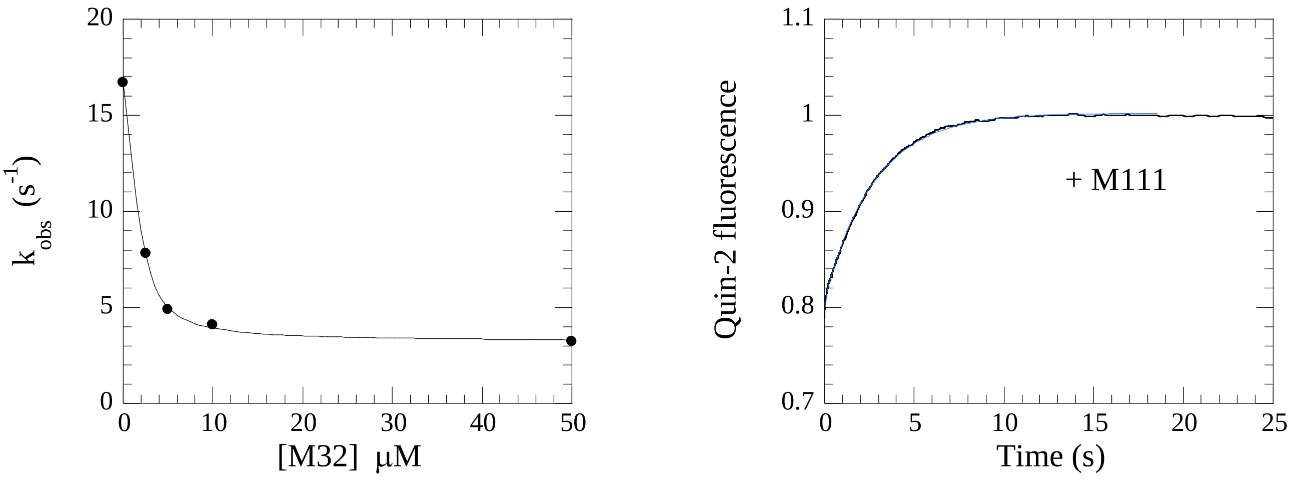

Fig. S3. (a) The dependence of the observed rate constant for Ca^{2+} dissociation from 10 μM S100A4 (when fit to a single exponential) as a function of [M32]. A fit to a quadratic binding equation yielded a K_d = 0.44 μM and a limiting k_{obs} at saturating M32 of 3.2 s^{-1}. The stoichiometry was 0.46 mole M32 per mole S100A4 monomer. Conditions were as described for Fig. 2c. (b) Dissociation of Ca^{2+} from 10 μM S100A4 in the presence of 5 μM M111 and 50 μM Ca^{2+} on mixing with 100 μM Quin-2 (conditions as described for Fig. 2d). A fit to a biphasic exponential yielded rate constants of 21 s^{-1} (6%) and 0.36 s^{-1} (94%); the former representing Ca^{2+} from the residual unbound S100A4. At 100 mM NaCl, the M111 construct remained soluble cf. the M200 construct which forms filaments on dissociation from S100A4 (Fig. 5d).

Fig. S4.

(a)

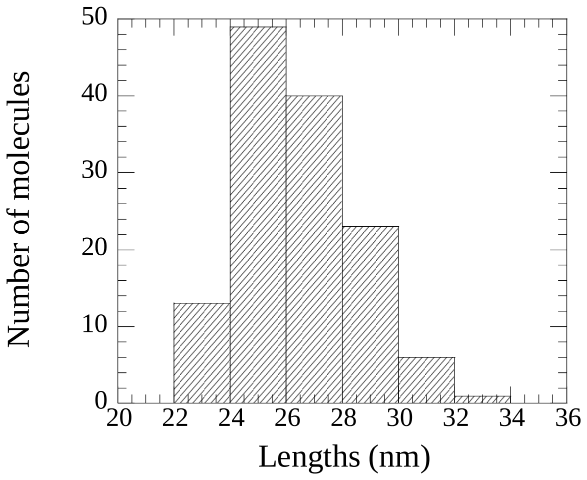

(b)

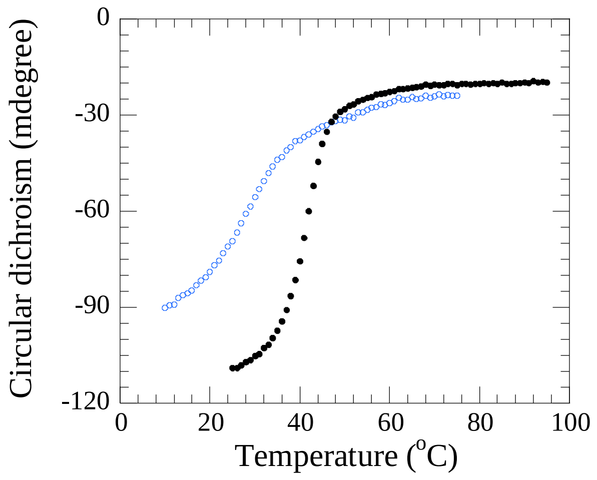

Fig. S4. (a) A histogram showing the distribution of the tail lengths measured from a rotary shadowed image M200. The distribution has a peak at 25 nm and the tail length was 26.4 nm ± 2.03 nm (mean ± S.D., n=132). (b) Melting of M200 (black solid symbols) and M111 (blue open symbols) as followed by the circular dichroism signal at 220 nm.

Fig. S5

(a) (b)

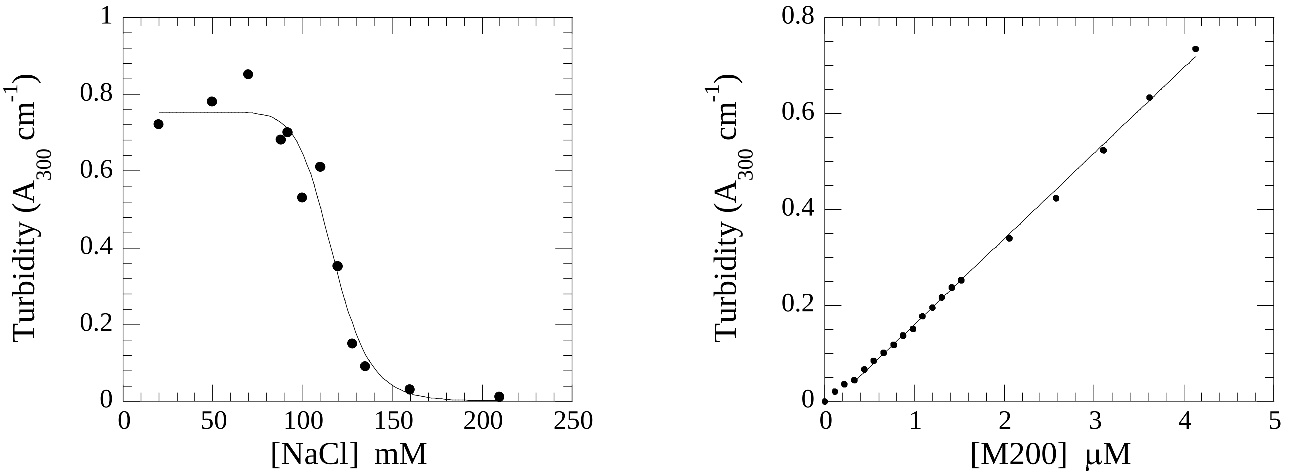

Fig. S5. (a) Turbidity of the M200 fragment (5 µM) as a function of [NaCl]. (b) Determination of the critical concentration of M200 in 100 mM NaCl, 10 mM Hepes, 0.1 mM Ca^{2+} at pH 7.5 and 20 ^{o}C. Aliquots of 1 or 5 µl of M200 were added to 1 ml buffer and the turbidity reading determined over a 30 s period. A linear fit to the data obtained above 0.3 µM indicated a critical concentration of 0.1± 0.04 µM.

Fig. S6.

(a)

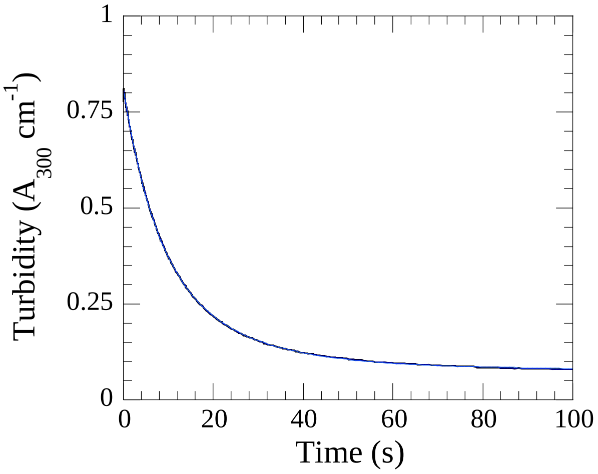

(b)

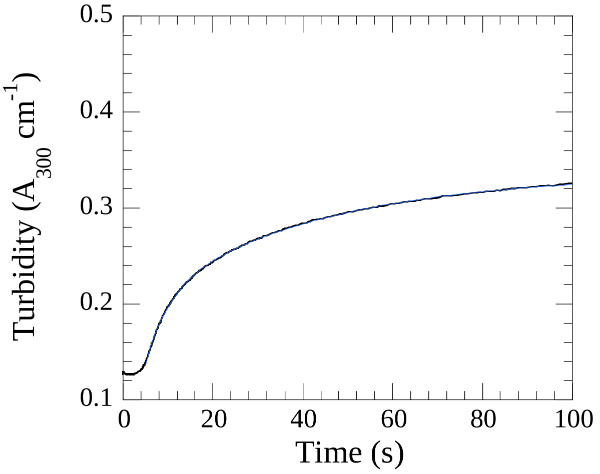

(c)

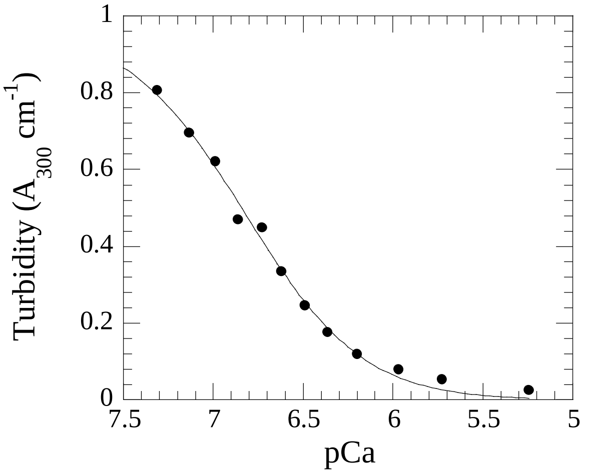

Fig. S6, (a) Dissociation of 5 μM M200 on mixing with 10 μM S100A4 measured using a stopped flow spectrophotometer in absorption mode. The fit to a double exponential yielded rate constants of 0.14 s^{-1} (56%) and 0.052 s^{-1} (44%). On a shorter time base, a lag phase of about 150 ms was revealed. (b) Formation of M200 filaments following S100A4 dissociation. One syringe contained 2.5 μM M200 + 5 μM S100A4 in 100 μM Ca^{2+}, while the other contained 0.5 mM EGTA. Following a 4 s lag, the biphasic rise in turbidity was fit to yield rate constants of 0.20 s^{-1} (56%) and 0.026 s^{-1} (44%). For (a) and (b) the buffer comprised

100 mM NaCl, 10 mM Hepes, 100 μM Ca^{2+} at pH 7.5 and 20 ^{o}C. (c) Ca^{2+} dependence of the turbidity of 5 μM M200 in the presence of S100A4 to yield an apparent K_d for Ca^{2+} binding of 0.14 μM. The buffer contained 100 mM NaCl, 20 mM Hepes, 1 mM Mg^{2+}, 1 mM EGTA and 1 μM Fura-2. Ca^{2+} was added in μl aliquots and the turbidity measured (using the 10 mm path length). The free Ca^{2+} was calculated from the ratio of the Fura-2 340 nm to 362 nm excitation peaks, assuming a K_d of 0.224 μM [1], measured between each addition of Ca^{2+} (using the 2 mm path length for excitation).

Fig. S7.

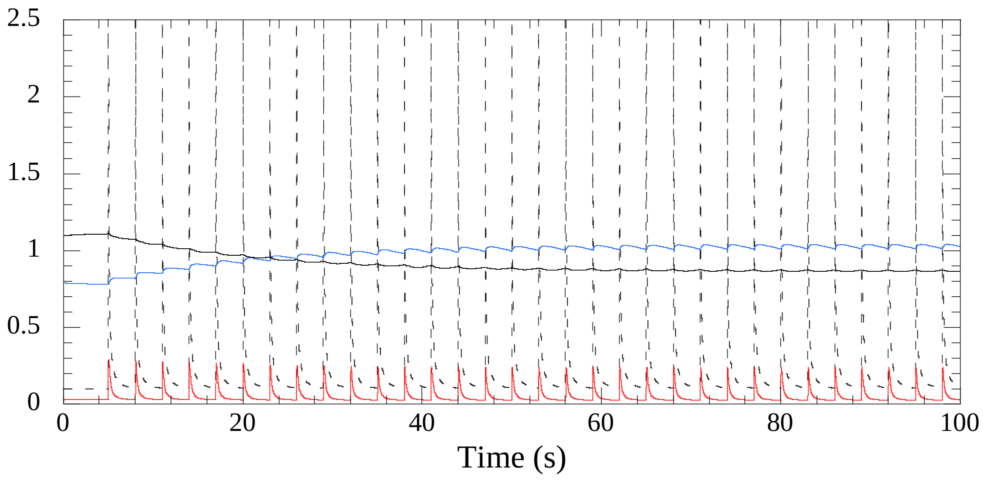

Fig. S7. Simulated response of the kinetic pathway (Scheme 1) to a train of Ca^{2+} waves (dashed line) with a rise time constant of 10 ms and a decay constant of 100 ms, repeating every 3 s (giving a mean [Ca^{2+}] = 0.22 μM) starting from a resting [Ca^{2+}] = 0.1 μM. Filament (black line), S100A4.Ca^{2+} (red line) and M.S100A4.Ca^{2+} (blue line). Note that filament disassembly barely responds to the individual Ca^{2+} transients, but does slowly respond to the mean elevated [Ca^{2+}]. Simulations were performed using the mechanism shown in Scheme 1 (main text) with k_0 = 0.1 $\mu M^{-1}s^{-1}$, k_{-0} = 100 s^{-1} (these are likely lower limits), k_1 = 4 $\mu M^{-1}s^{-1}$, k_{-1} = 16 s^{-1}, k_2 = 10 $\mu M^{-1}s^{-1}$, k_{-2} = 0.03 s^{-1}, k_3 = 25,000 $\mu M^{-1}s^{-1}$, k_{-3} = 0.3 s^{-1}. In addition, myosin filament formation was modelled, for simplicity, as a cooperative transition 5M ↔M_5 to give a system in which the free M remained around 0.1 μM for the total M concentration range explored in the simulations (k_f = 1000 $\mu M^{-4}.s^{-1}$, k_r = 0.01 s^{-1}). The value 5 for the filament size was selected as a compromise between the likely higher degree of cooperativity involved in filament formation and ease of computation. Further interactions included S100A4 + M_5 ↔ S100A4.M_5 with k_f = 10 $\mu M^{-1}s^{-1}$ and k_r = 30 s^{-1} (i.e. K_d = 3 μM for binding to filament) and M.S100A4.Ca^{2+} + 4M ↔ S100A4.M_5 with k_f = 100 $\mu M^{-4}.s^{-1}$ and k_r = 1 s^{-1}. Values for these parameters were selected to give a response comparable to those observed *in vitro* for M200 and to maintain

the overall thermodynamic balance. The same parameters were used for the simulations carried out in Fig 10a,b (main text), apart from the free [Ca^{2+}] .

References

1. Grynkiewicz, G., Poenie, M. & Tsien, R. Y. (1985). A new generation of Ca2+ indicators with greatly improved fluorescence properties. *J Biol Chem* **260**, 3440-50.
